# Self-healing mixed matrix membranes containing metal–organic frameworks†

**DOI:** 10.1039/d2sc04345a

**Published:** 2022-09-29

**Authors:** Prantik Mondal, Seth M. Cohen

**Affiliations:** Department of Chemistry and Biochemistry, University of California La Jolla San Diego California 92093 USA scohen@ucsd.edu

## Abstract

Mixed-matrix membranes (MMMs) provide a means to formulate metal–organic frameworks (MOFs) into processable films that can help to advance their use in various applications. Conventional MMMs are inherently susceptible to craze or tear upon exposure to impact, cutting, bending, or stretching, which can limit their intended service life and usage. Herein, a simple, efficient, and scalable *in situ* fabrication approach was used to prepare self-healing MMMs containing Zr(iv)-based MOFs. The ability of these MMMs to self-heal at room temperature is based on the reversible hydrolysis of boronic-ester conjugates. Thiol–ene ‘photo-click’ polymerization yielded robust MMMs with ∼30 wt% MOF loading and mechanical strength that varied based on the size of MOF particles. The MMMs could undergo repeated self-healing with good retention of mechanical strength. In addition, the MMMs were catalytically active toward the degradation of the chemical warfare agent (CWA) simulant dimethyl-4-nitrophenyl phosphate (DMNP) with no change in activity after two damage-healing cycles.

## Abstract 2

Self-healable mixed-matrix membranes (MMMs) are described with adjustable mechanical strength, dynamic covalent chemistry, and metal–organic frameworks (MOFs) that degrade a toxic chemical warfare agent (CWA) simulant.

## Graphical abstract

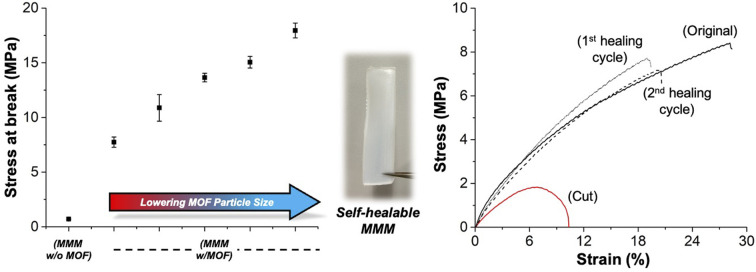

## Introduction

Metal–organic frameworks (MOFs) are a class of inorganic–organic hybrid materials that are crystalline and porous, consisting of metal ions or metal oxide nodes (secondary building units, SBUs) bridged by multitopic organic ligands.^1–3^ Because of their stable and tunable porosity, accessible pores, and rich chemical and structural diversity, MOFs are increasingly the focus of research for applications in gas storage, separations, catalysis, sensing, and polymeric membranes.^2,4–9^ The synergism of the organic and inorganic components improves the crystallinity and structural robustness of MOFs;^3^ however, their particulate form (brittle and fragile macroscopic crystals or microcrystalline powders) restricts the processability of MOFs for certain applications.^10^ MOF-based polycrystalline membranes have been synthesized, but their synthetic protocols are tedious, and only a few MOFs have been reported in this form.^7,11^

Alternatively, the fabrication of free-standing, flexible, and mechanically durable mixed-matrix membranes (MMMs) offers an alternative approach to formulating MOFs.^12–14^ MMMs are assembled by blending polymer materials with MOFs, where the MOF acts as a ‘filler’. Such polymeric composites can display superior mechanical strength (*e.g.*, high stiffness and rigidification) while preserving favourable characteristics of the MOF (*e.g.*, sorption, catalysis, *etc.*). To date, MMMs with MOFs have been prepared using several polymeric materials like poly(ethylene-*co*-vinyl acetate) (EVA), poly(vinylidene fluoride) (PVDF), poly(ethylene oxide) (PEO), styrene–butadiene (SBS) copolymers, polyurethanes, *etc.*^15–24^ These MMMs have been investigated for applications in sensing,^25^ separation of dyes and toxic chemicals,^16,17^ and other technologies. Not all polymer–MOF combinations are suitable for forming MMMs, as MOF particles often fail to combine well with the polymer matrix and can suffer from particle aggregation that disrupt the function and mechanical strength of the resulting MMM.^26–28^ The uniform and aggregation-free dispersion of MOF particles in a polymer matrix play an essential role in improving the mechanical strength of composites.^29^ The fabrication of polymeric composites using *in situ* polymerization may improve the dispersion homogeneity and distribution of the MOF fillers in the polymer matrix.^30–32^

Based on the available reports^33,34^ on polymer composites (using inorganic particulates as fillers, *e.g.*, micro or nano-silica, glass, aluminium oxide, magnesium hydroxide, calcium carbonate, carbon nanotubes, *etc.*), their mechanical strength largely relies on filler particle size, as well as other factors such as filler loading and distribution in the polymer matrix. MMMs with higher MOF loading (wt%) often exhibit reduced flexibility (due to high brittleness) and lack polymer-filler compatibility, significantly deteriorating their physical strength.^29,35^ Although several reports address the effect of particle size of other inorganic particulates on the tensile strength of their corresponding polymer MMM composites, there have been no investigation dealing with the influence of MOF particle size on the strength of MMMs. As such, the understanding of the effect of differently sized MOF particles on the mechanical strength of their corresponding polymer MMM composites is quite understudied.

MMMs prepared from most polymer materials are not refractory to mechanical insults that might that cause crazing (fine cracks on the surface), cracking, or fracture. Consequently, the physical integrity of the MMMs can be compromised, ultimately reducing their intended service life.^36^ Following the landmark reports of White *et al.*^37^ and Wudl *et al.*,^38^ different dynamic approaches have been exploited to produce self-healing polymers and materials.^39^ Reversible, exchangeable chemical reactions have been used in numerous accounts of self-healable polymer materials;^40^ however, the combining of dynamic covalent chemistry with MOFs, *e.g.*, for generating self-healable MMMs, has not been reported. Indeed, while numerous healable polymers have been developed, the synthesis of self-healable MMMs has remained largely unexplored. Making MMMs self-healable could improve the lifetimes and mechanical durability of these membranes and further extend the utility and usefulness of these composite materials.

Our continued interest in preparing MMMs for new applications^9,12^ prompted the work reported herein. A simple and scalable route is described to prepare self-healable MOF-based MMMs using thiol–ene ‘photo-click’ polymerization and reversible hydrolysis of boronic-ester conjugates. Unlike the many prior MMM synthetic protocols (which involve the physical mixing of the polymers as a ‘binder’ with pre-formed MOF filler suspensions), the procedure reported here involves the *in situ* fabrication of MMMs by ‘photo-click’ polymerization of a suspension of finely dispersed MOF particles in the monomer mixture (so-called postsynthetic polymerization, PSP).^41^ The reversibility of boronic-ester crosslinks is activated by moisture (85% humidity) or liquid water, which endows these MMMs with dynamic and self-healing features that can proceed at room temperature. The healable MMMs show a considerable recovery (>75%) in their tensile strength even after two damage-healing cycles. As expected, the MOF particle features (*e.g.*, size, shape, crystallinity) remain unperturbed upon healing. Interestingly, the mechanical behaviour of the MMMs depends upon the size of the MOF particles. A systematic investigation of the structure–property relationships in these MMMs was thus performed as a function of MOF particle size. As demonstrated *via* tensile analysis, the MMMs prepared using smaller MOF particles exhibited better stiffness and strength than those prepared with larger particles. The ability of these MMMs to degrade a chemical warfare agent (CWA) simulant was retained even after multiple healing cycles. To the best of our knowledge, there are no reports describing the systematic influence of MOF particle size on the mechanical strength of the corresponding MMMs and the exploitation of dynamic covalent chemistry to generate ambiently healable, catalytically active MMMs.

## Results and discussion

### Synthesis and characterization of MOFs

Different Zr(iv)-based MOFs were employed for this study. MOFs were designated as MOF_*X*_, where *X* = average particle edge length (nm) as measured by scanning electron microscopy (SEM, Fig. S1†). Using acetic acid (UiO-66_330_, UiO-66-NH_2-170_) or formic acid (MOF-808_140_) as modulators allowed for the preparation of MOF particles of varying sizes and topologies. MOF-808_140_ was synthesized by combining zirconyl chloride octahydrate (ZrOCl_2_·8H_2_O) with 1,3,5-benzenetricarboxylic acid (H_3_btc) using formic acid as the modulator at 110 °C for 48 h.^42^ UiO-66_330_ and UiO-66-NH_2-170_ were synthesized using terephthalic acid (H_2_bdc) and 2-aminoterephthalic acid (H_2_bdc–NH_2_) as the organic ligands, acetic acid as the modulator, and DMF as a solvent with heating at 120 °C for 24 h. The bulk crystallinity of the MOF was assessed by powder X-ray diffraction (PXRD), which in all cases closely resembled the simulated patterns (Fig. S2†). The MOFs were digested in dilute acid (see ESI† for details) and analysed *via*^1^H NMR analysis to confirm the presence of the organic ligands after the MOF formulation (Fig. S3†). HR-ESI-MS analysis of the digested Zr-MOFs showed only the parent ligand base peak in good agreement with the expected [M − H]^−^ or [M + H]^+^ ion of the corresponding ligand mass (Fig. S4†).

### Synthesis of MMMs

**Fig. 1:**
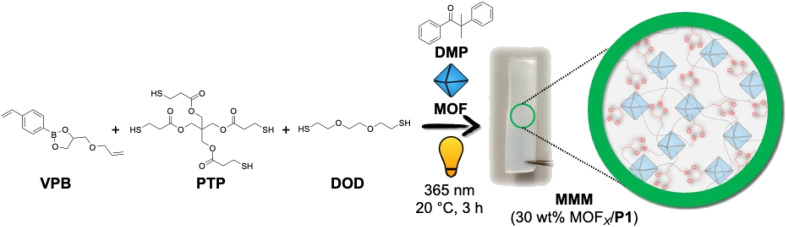
Scheme for synthesizing P1-based MMMs with 30 wt% MOF_*X*_-loading using thiol–ene ‘photo-click’ polymerization.

Fabrication of self-healing MMMs was achieved using thiol–ene ‘photo-click’ polymerization that is additive-free, tolerant to moisture and air, and can be readily conducted using a wide range of commercially available monomers under mild reaction conditions.^43–46^ The general preparatory method (see ESI for details, Table S1†) of the MMMs includes a fine dispersion of the MOF particles in ethyl acetate, to which the mixture of monomeric components and photo-initiator was added. After gently vortexing the reaction components, the suspension was cast into a Teflon mould and transferred to a UV chamber, where the thiol–ene polymerization was conducted by irradiating the mixture at 365 nm for 3 h. More specifically, the aromatic divinyl monomer containing dynamic boronic-ester conjugate (4-((allyloxy)methyl)-2-(4-vinylphenyl)-1,3,2-dioxaborolane, VPB) was synthesized and subsequently polymerized *via* thiol–ene ‘photo-click’ chemistry with aliphatic di-thiol (2,2'-(ethane-1,2-diylbis(oxy))bis(ethane-1-thiol), DOD) and tetra-thiol (2,2-bis(((3-mercaptopropanoyl)oxy)methyl)propane-1,3-diyl bis(3-mercaptopropanoate), PTP) under UV irradiation (365 nm) in the presence of 2,2-dimethoxy-2-phenylacetophenone (DMP) as a photo-initiator and different Zr(iv)-based MOFs (*e.g.*, MOF-808_140_, UiO-66_330_, and UiO-66-NH_2-170_), to fabricate MMMs with boronic-ester linkages (Fig. 1). Ethyl acetate was used as the solvent to disperse the MOFs in which the reaction components were miscible and stable.

The boronic-ester-based crosslinked network (without MOF particles) obtained after the thiol–ene photo-polymerization of VPB, DOD, and PTP was designated as P1. P1 is a thermoset, and as such, the molecular weight of the polymer network cannot be determined *via* conventional methods (*e.g.*, gel-permeation chromatography, NMR) because of the insolubility of the material. Pure P1 was a soft material, but its formulation into MMMs with MOF_*X*_ particles significantly increased the rigidity of the membranes. For all the MMMs, the molar equivalence of VPB, PTP, and DOD was maintained at 5:1:3, using 1 wt% DMP (with respect to the total amount of VPB, PTP, and DOD) and 30 wt% MOF_*X*_ (with respect to the total amount of VPB, PTP, DOD, and DMP). The boronic-ester-based MMMs were designated as 30 wt% MOF_*X*_/P1, where P1 indicates the boronic-ester polymer and MOF_*X*_ indicates the MOF_*X*_ particles used. In parallel experiments, divinylbenzene (DVB) was used instead of VPB under similar polymerization conditions to generate control MMMs containing polymers that cannot self-heal (Fig. S5†). The crosslinked network (without MOF particles) obtained after the thiol–ene photo-polymerization of DVB, DOD, and PTP was designated as P2, and its corresponding MOF-based membranes are represented as 30 wt% MOF_*X*_/P2.

Except for UiO-66-NH_2-170_, the photo-polymerization protocol generated highly crosslinked, free-standing, and flexible MMMs (Fig. S6†). The use of UiO-66-NH_2-170_ did not lead to a free-standing MMM; instead, a viscous, yellowish mass was obtained (Fig. S6†), indicating the polymerization was incomplete. This outcome was attributed to *in situ* deprotonation of the thiol monomers by the MOF amines (from UiO-66-NH_2-170_), which produces a thiolate anion that reacts with thiyl radicals and inhibits polymerization. According to Bowman and coworkers, the combination of thiolate anion and thiyl radicals generates two-sulfur three-electron bonded disulfide radical anionic (DRA) species that sequester thiol radicals and retard polymerization.^47,48^ Inhibition of polymerization increases with increasing feed content (mol%) of amines with respect to the thiol. In the MMM polymerization, the 30 wt% UiO-66-NH_2-170_ (0.3 mmol, 13 eq.) greatly exceeds the thiol monomer content (PTP, 0.023 mmol, 1 eq.), which is more than sufficient to completely inhibit polymerization. Further evidence of polymerization inhibition was provided by FTIR analysis of the final mixture, which showed the presence of unreacted –SH groups (Fig. S7†). For further confirmation, MMM fabrication using a much lower content of UiO-66-NH_2-170_ (0.7 wt%, 5 μmol, 0.25 eq.) with respect to PTP (0.023 mmol, 1 eq.) did result in a free-standing polymer film (0.7 wt% UiO-66-NH_2-170_/P1; Fig. S7 and S8†).

### Characterization of MMMs

The completion of the thiol–ene ‘click’ polymerization was verified by ATR-FTIR analysis of the MMMs (Fig. S9†), which shows the difference in the intensity of stretching frequency of thiols (
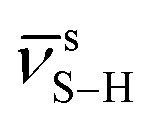
) at ∼2560 cm^−1^ before and after curing reaction components to generate 30 wt% MOF_*X*_/P1. The thiols are entirely consumed *via* the thiol–ene reaction with the vinyl units of VPB, as evidenced by the complete disappearance of the absorption peak of –SH in all the post-cured materials (except with UiO-66-NH_2-170_), suggesting that the polymerization was quantitative.^49^ The absence of excess thiols is essential to avoid disulfide bond formation that could reduce the healing rate at room temperature, as disulfide exchange generally requires higher temperatures (≥60 °C).^50^

Thermogravimetric analysis (TGA) was performed to determine the final MOF loading in the MMMs based on the weight loss corresponding to P1 (at ∼300–400 °C) and the MOFs (∼400–450 °C) (Table S2 and Fig. S10†). The TGA data indicates that the loading of all the MOFs was consistent with the theoretical fraction of MOFs used during MMM synthesis, suggesting the quantitative incorporation of MOF particles. TGA analysis performed with the control MMMs (P2-based MMMs) also showed a good correlation between the experimental MOF-loading and the expected amount (Table S2 and Fig. S10†).

**Fig. 2:**
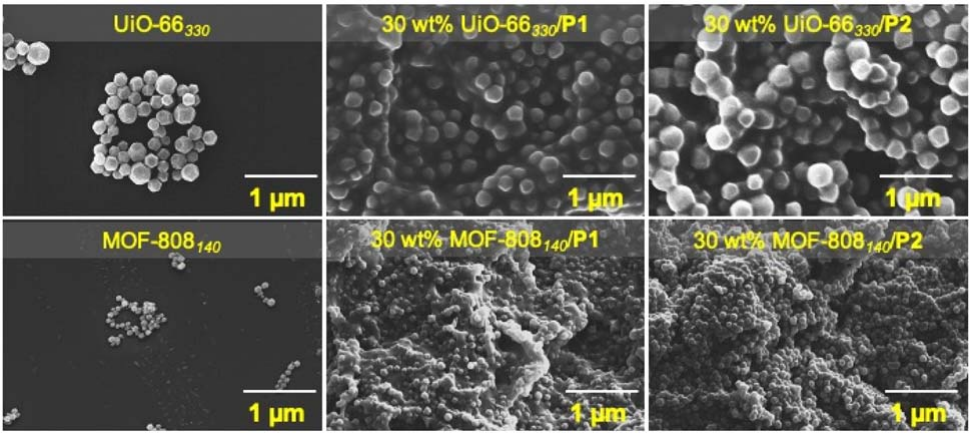
Top: SEM images of UiO-66_330_ and the cross-sectional surface of the corresponding P1-based and P2-based MMMs. Bottom: MOF-808_140_ and the cross-sectional surface of the corresponding P1-based and P2-based MMMs (1 μm scale bars).

Following the assessment of the MOF composition in the MMMs, the characterization of the MOF after membrane fabrication was examined. The surface morphology of the MMMs was characterized *via* SEM. SEM images of the cross-section, top, and bottom surfaces of the P1-based MMMs showed uniform (and aggregation-free) dispersion of the MOF particles throughout the polymer matrix (Fig. 2).

The MOF particles in P2-based MMMs were also distributed evenly throughout the polymer matrix (Fig. S11†). The crystallinity of the MOF particles was retained in all the MMMs, as evidenced by PXRD (Fig. S12†). The MMMs were non-porous, as evidenced by their nitrogen (N_2_) gas sorption and the calculated Brunauer–Emmett–Teller (BET) surface areas (Table S3†). The N_2_ adsorption analysis at 77 K may have immobilized the polymer chains resulting in MOF pore blockage, resulting in the low observed BET surface area of the MMMs.^51,52^

### Tensile analysis of MMMs

**Fig. 3:**
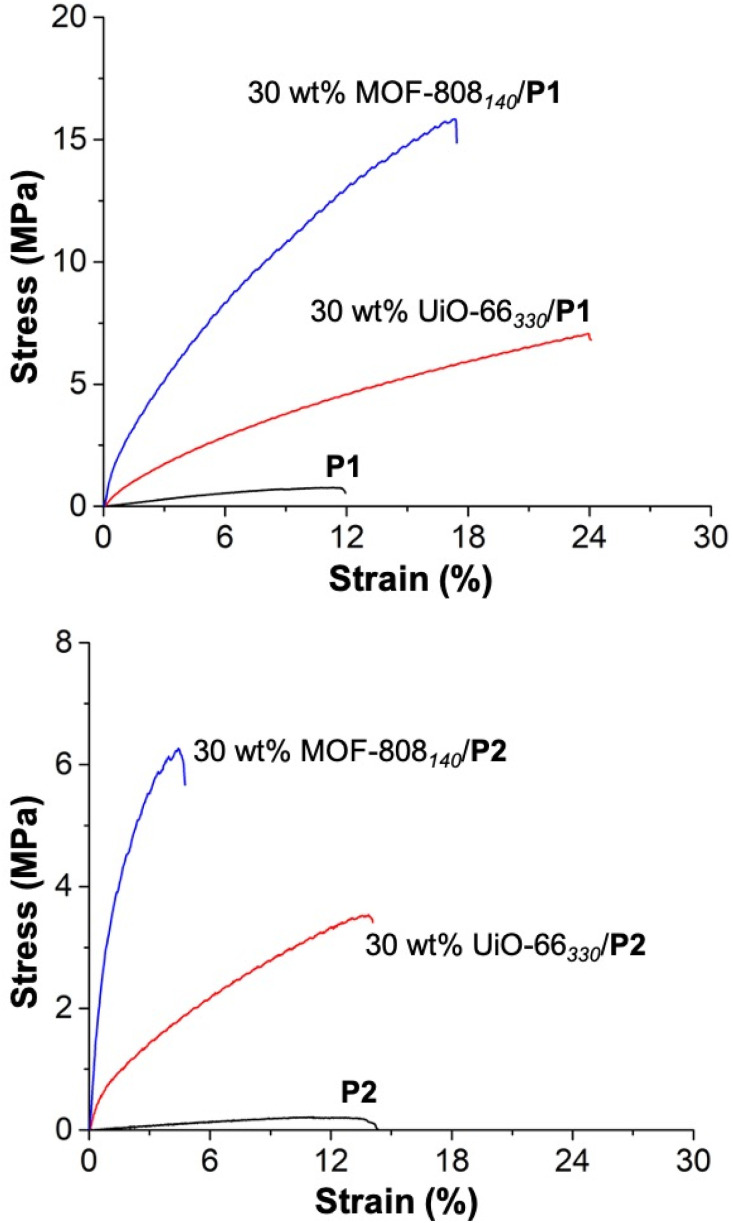
Evaluation of the mechanical strength of P1 and P1-based MMMs (top) and P2 and P2-based MMMs (bottom) *via* tensile analysis obtained by the uniaxial deformation of the tensile bar until failure (at 10 mm min^−1^ strain rate and 20 °C). Note the different scales of the *y*-axis between the two plots.

The mechanical strength of all the MMMs was evaluated by tensile analysis in terms of tensile stress at break (*σ*_b_), defined as the maximum stress an MMM can sustain under uniaxial tensile loading before its failure.^34^ For consistency, the membranes were dried at room temperature under a vacuum for 24 h, and the tensile testing was then performed immediately (within ∼1–2 min) after their removal from vacuum. Fig. 3 shows the tensile curves of the membranes, and the results are summarized in Table S4.† The tensile analysis unambiguously shows the expected influence of incorporating MOFs, which significantly improved the tensile strength of the MMMs (over pure P1 and P2). Compared to pure P1, the *σ*_b_ of the P1-based MMMs (30 wt% MOF-808_140_/P1 and 30 wt% UiO-66_330_/P1) substantially increased with 30 wt% MOF loading. In traditional inorganic–organic hybrid polymeric composites (*e.g.*, using metal oxides), the homogeneous dispersion of crystalline fillers in the amorphous polymer matrix is known to improve the compatibility between the two components and mechanical properties of the MMM.^30–32^ As evidenced by the SEM analysis (Fig. 2), the MOF–polymer compatibility promoted the agglomeration-free and uniform distribution of crystalline MOF particles throughout the bulk of the P1 matrix, resulting in a significant increase in MMM performance before failure.^53^

The tensile strength of P2-based MMMs (30 wt% MOF-808_140_/P2 and 30 wt% UiO-66_330_/P2) was somewhat different from P1-based MMMs. The *σ*_b_ of P2-based MMMs was improved (over pure P2); however, the changes were smaller compared to the P1-based MMMs (Fig. 3). This difference is rationalized based on the following arguments. First, the difference in the chemical nature of aromatic-based divinyl monomers, *i.e.*, VPB and DVB, play a crucial role in deriving the polymer networks bearing distinct tensile features. Cyclic units (*e.g.*, cyclic acetal) are commonly incorporated into polymers such as polycarbonates and carbohydrates to impart stiffness into the macromolecular chains that remarkably increase their physical strength.^54,55^ Tensile analysis of P1 and P2 showed P1 (0.71 ± 0.014 MPa) exhibited a larger *σ*_b_ than P2 (0.21 ± 0.02 MPa). Thus, the presence of an additional (boronic-ester) cyclic unit in VPB perhaps improves the stiffening characteristics of the P1-based polymer networks, as evidenced by their corresponding higher *σ*_b_ compared to P2-based polymer materials. Second, in a chemically cross-linked polymer, the entanglement of macromolecular chains acts as the physical cross-link domains that improve stiffness,^56,57^ preferably relies on the length of a polymer chain (*e.g.*, conventional elastomers).^58^ VPB is a longer monomer than DVB, thus leading to a polymer with a longer repeat unit, which presumably results in a higher degree of chain entanglement in P1- *versus*P2-based polymer materials. The *σ*_b_ gradually increases with the extent of entanglement, as that polymer comprises more (entwined) polymer chains to withstand the tension. Hence, the increased chain entanglements likely improved the stiffness (and thus *σ*_b_) of P1-based polymer materials over their P2-based materials.

More interestingly, despite identical loadings (30 wt%) of MOFs in these MMMs, a distinct increase in *σ*_b_ (16.6 ± 0.56 MPa) was observed with 30 wt% MOF-808_140_/P1 over 30 wt% UiO-66_330_/P1 (7.74 ± 0.47 MPa). A similar trend was observed with the P2-based MMMs, where the *σ*_b_ of 30 wt% MOF-808_140_/P2 (5.75 ± 0.36 MPa) was higher than 30 wt% UiO-66_330_/P2 (*σ*_b_ = 3.61 ± 0.05 MPa). Available reports on polymeric composites using inorganic particle fillers suggest that the smaller particle size of MOF-808_140_ compared to UiO-66_330_ may play a role in the observed differences in *σ*_b_, which is directly correlated with the strength and stiffness of the MMMs.^59,60^ Typically, the non-bonding (physical) interaction between the polymer chains and inorganic particles increases with the decreasing size of the filler particles. Because of the higher surface-to-volume ratio and enlarged particle-to-matrix interface area, the smaller particles can bind the polymer segments more firmly, significantly rigidifying the polymer composites.

**Fig. 4:**
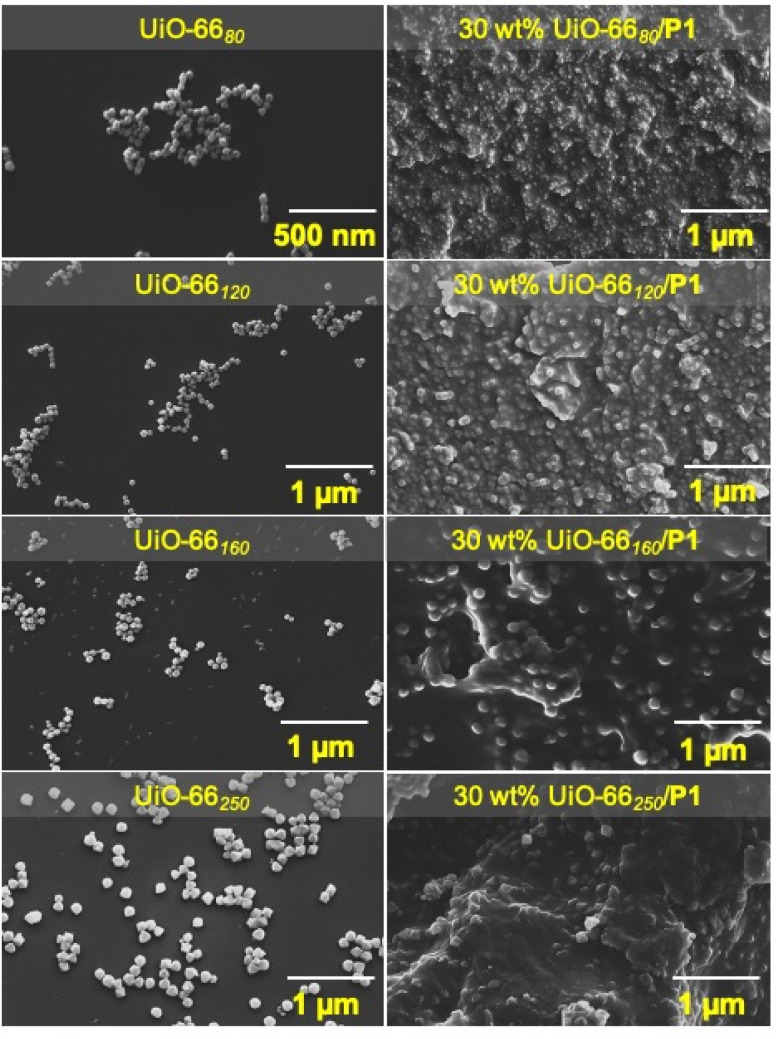
Left: SEM images of UiO-66_80_, UiO-66_120_, UiO-66_160_, and UiO-66_250_. Right: cross-sectional image of corresponding P1-based MMMs (500 nm scale bar for UiO-66_80_ and 1 μm scale bar for all other images).

To further elucidate the influence of particle size and chain entanglements on the strength of MMMs, UiO-66 MOFs of different sizes were synthesized (UiO-66_80_, UiO-66_120_, UiO-66_160_, and UiO-66_250_; synthetic details are available in the ESI) and subsequently used in MMM fabrication. Using an identical methodology as described above, 30 wt% UiO-66_80_/P1, 30 wt% UiO-66_120_/P1, 30 wt% UiO-66_160_/P1, and 30 wt% UiO-66_250_/P1 MMMs (and their P2 analogues) were synthesized. The completion of the polymerization reaction was confirmed by the FTIR analysis (Fig. S13†). SEM images of the cross-section, top, and bottom sides of the MMM showed the uniform (and aggregation-free) dispersion of the MOF particles throughout the P1 matrix (Fig. 4, S14 and S15†). PXRD analysis confirmed that the crystallinity of the MOF particles was retained after MMM fabrication (Fig. S16†). The TGA analysis showed that the experimental loading of MOF in all the MMMs was in good accordance with the theoretical value (Table S2, Fig. S17 and S18†).

**Fig. 5:**
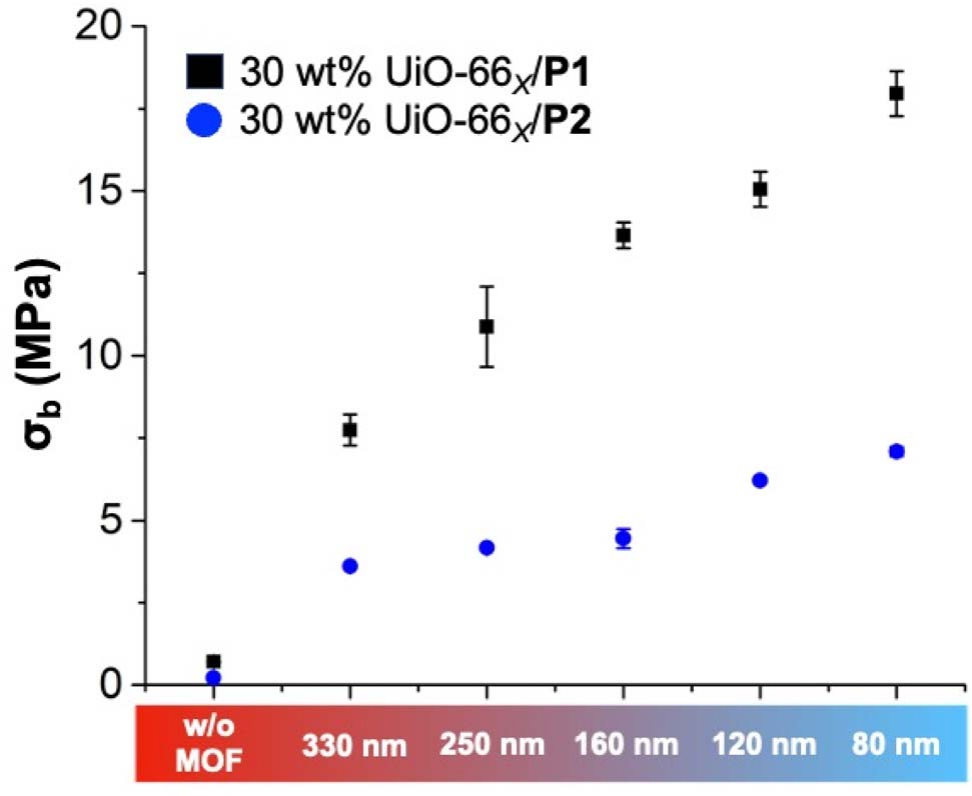
Measurements of *σ*_b_ from P1- and P2-based MMMs without MOFs and with UiO-66_*X*_ (*X* = 80 nm, 120 nm, 160 nm, 250 nm, 330 nm) particles, illustrated *via* tensile analysis obtained by the uniaxial deformation of the tensile bar until failure (at 10 mm min^−1^ strain rate and 20 °C).

Applying the same experimental protocol, the mechanical strength of the MMMs was investigated *via* tensile analysis. Intriguingly, the *σ*_b_ of the P1-based MMMs remarkably improved as the size of UiO-66_*X*_ MOF particles decreased. Noticeably, 30 wt% UiO-66_80_/P1 exhibited the highest *σ*_b_ (17.95 ± 0.67 MPa) compared to the other P1-based MMMs. Fig. 5 shows an increasing linear trend of *σ*_b_ of the P1-based MMMs with lowering the UiO-66_*X*_ particle size. This indicates that despite similar MOF loading (30 wt%) in all the MMMs, their tensile strength primarily relied on the MOF particle size. For a given particle loading (in this case, 30 wt%), smaller-sized MOF particles likely possess a higher total surface area, allowing more polymer chains to bind, entwine, and improve the degree of chain entanglement. This also specifies that the tensile strength of the MMMs enhanced with an increased surface area of the filled MOF particles. Unlike the smaller-sized MOF particles, the macromolecular chains possibly cannot form a desirable degree of entanglement with larger MOF particles due to reduced surface-to-volume ratio and particle-to-matrix interface area, reducing the overall stiffness of their corresponding MMMs. The results are in good accordance with the existing reports on polymer composites using differently sized traditional inorganic fillers.^33,34^ Despite using different Zr-based MOFs, the tensile strength of 30 wt% UiO-66_160_/P1 (*σ*_b_ = 13.65 ± 0.38 MPa) and 30 wt% MOF-808_140_/P1 (*σ*_b_ = 16.6 ± 0.56 MPa) was almost the same, likely due to the similar particle sizes, further suggesting the role of smaller-sized MOF particles in relatively improving the strength of the MMMs. The *σ*_b_ of the P2-based MMMs using UiO-66_*X*_ particles displayed a similar increasing trend to P1-based MMMs. To the best of our knowledge, the current report is the first description on the effects of MOF fillers on the mechanical strength of MMMs that explicitly considers different MOFs (UiO-66 and MOF-808) and MOF particle sizes. Although several MOFs and inorganic fillers have been used to prepare MMMs, no reports describe the effect on MMM mechanical strength for the types and size fillers presented in this work.

### Dynamic behaviour of MMMs

Self-healing polymer materials exhibit dynamic or adaptable characteristics under the influence of external stimuli, which are based on component exchange or reorganization *via* reversible chemical reactions. A model study was performed (using P1 and P2) *via* tensile analysis to confirm the dynamic behaviour of P1 (Fig. S19†). As expected, unlike P2, the tensile strength of P1 was substantially reduced (with increased elasticity) after aging under 85% humidity for 24 h. This indicates that a portion of boronic-ester conjugates underwent hydrolysis upon exposure to high humidity, reducing the cross-link density of P1.

**Fig. 6:**
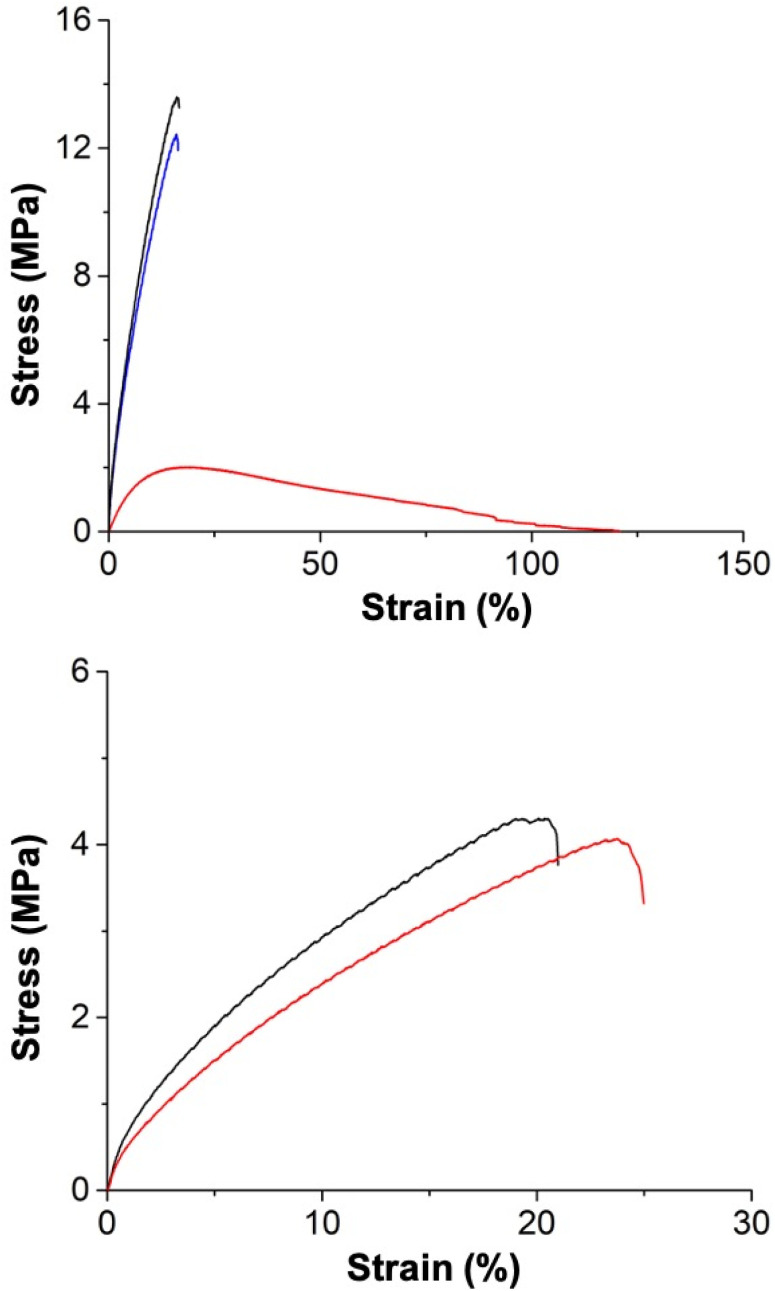
Mechanical testing of the dynamic behaviour of 30 wt% UiO-66_160_/P1 (top) and 30 wt% UiO-66_160_/P2 (bottom) MMMs by observing changes in their original tensile strength after exposing to 85% humidity for 24 h and subsequently drying at room temperature under a vacuum. Black traces are the original samples, red traces are after being aged at 85% humidity for 24 h, and blue traces are after re-drying (at 10 mm min^−1^ strain rate and 20 °C).

Tensile analysis was performed with pristine and aged (under 85% humidity) samples of 30 wt% UiO-66_160_/P1 (as a representative example) to investigate changes in mechanical performance. After aging at 85% humidity for 24 h, the P1-based MMM exhibited significantly reduced *σ*_b_, indicating the substantial loss of cross-link density (Fig. 6). The moisture sensitivity of boronic-ester conjugates triggered the P1-based MMM to absorb atmospheric water that hydrolysed a predominant proportion of the boronic-ester crosslinks, leading to network disorganization. Also, the strain value of the aged P1-based MMM increased, indicating the increase in the polymeric elasticity and mobility of polymer chains due to the reduction of the network density. The stress–strain behaviour of the P1-based MMM was almost entirely restored after drying the aged material (Fig. 6). A control tensile analysis was also performed with a P2-based MMM (30 wt% UiO-66_160_/P2), which did not show any diminution in mechanical performance (Fig. 6), underlining the significance of reversible conjugates in making P1-based MMM dynamic.

### Self-healing of MMMs

**Fig. 7:**
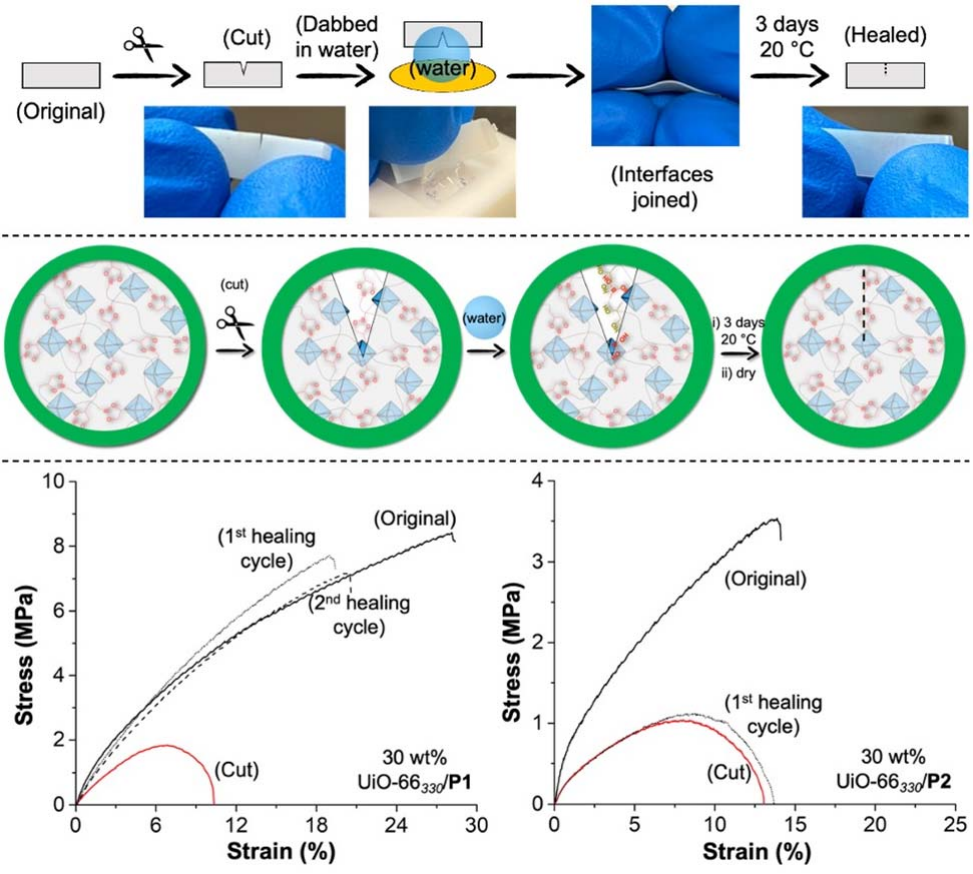
Top: scheme of cut-healing of 30 wt% UiO-66_330_/P1. Middle: proposed mechanism of cut-healing of P1-based MMMs. Bottom: stress–strain behaviour of cut-healed 30 wt% UiO-66_330_/P1 (left) and 30 wt% UiO-66_330_/P2 (right). Adsorption assay (monitored at 407 nm).

Driven by the reversibly exchangeable characteristics of the boronic-ester conjugates, the P1-based MMMs were dynamic under 85% humidity conditions, as demonstrated *via* the tensile analysis. A cut-healing protocol experiment was performed to examine if the boronic-ester reversibility could be implemented for healing damaged MMMs (Fig. 7). For this purpose, a rectangular-shaped polymeric specimen (with a dimension like the samples used for tensile analysis) was partially cut at the middle (cut size ∼1.8 ± 0.2 mm) using a scissor. The cut portion was wetted at the interface with a few drops of water for ∼15 s and subsequently joined by hand and held for ∼1 min before storing the materials to heal under ambient atmospheric conditions for 3 d. The cut interfaces of P1-based MMMs immediately adhered after wetting with water and reconnected rapidly (within minutes) under ambient conditions. Ultimately, the MMMs were dried at room temperature under a vacuum for 24 h to completely heal and restore membrane integrity.

The self-healing efficiency of the healed P1-based MMMs was evaluated by performing tensile testing experiments on healed MMMs and analysing the recovery of their corresponding *σ*_b_ value (with respect to their pristine and cut forms).

After wetting with water, reconnecting, and storing the cut MMMs for 3 d under ambient conditions, a considerable recovery (>75%) of tensile strength was observed for all MMMs (Fig. S20 and S21†), even after the second cutting-healing cycle. After healing, the crystallinity and shape of the MOF particles in the MMMs were preserved, as evidenced by the PXRD and SEM analyses (Fig. S22 and S23†). To ensure that the reversibility of the boronic-ester units played an integral role in healing the MMMs, a control cut-healing experiment was also performed with 30 wt% UiO-66_330_/P2; as expected, the 30 wt% UiO-66_330_/P2 failed to exhibit healing under identical experimental conditions (Fig. 7).

Based on the excellent healing efficiency of 30 wt% MOF_*X*_/P1, a qualitative experiment was also performed to check self-healing at a macroscopic level. In this experiment, the film (of 30 wt% UiO-66_330_/P1) was entirely cut into two halves, followed by adding a few drops of water on the interfaces and subsequently joining. Again, the cut halves instantly adhered and became inseparable after 5 min (ESI Movie S1†). After 4 d (under ambient atmosphere), the scar on the surface disappeared entirely (Fig. S24†).

### DMNP degradation by MMMs

Zr(iv)-based MOFs can be quite active for the catalytic degradation of nerve agent simulants, such as dimethyl 4-nitrophenyl phosphate (DMNP).^61^ Use of these MOFs in real-world applications (*e.g.*, as functional textiles) is enabled by integrating these fine powders into the polymer matrices, such as MMMs. As such, the MMMs were screened for catalytic activity against DMNP to assess their activity.

**Fig. 8:**
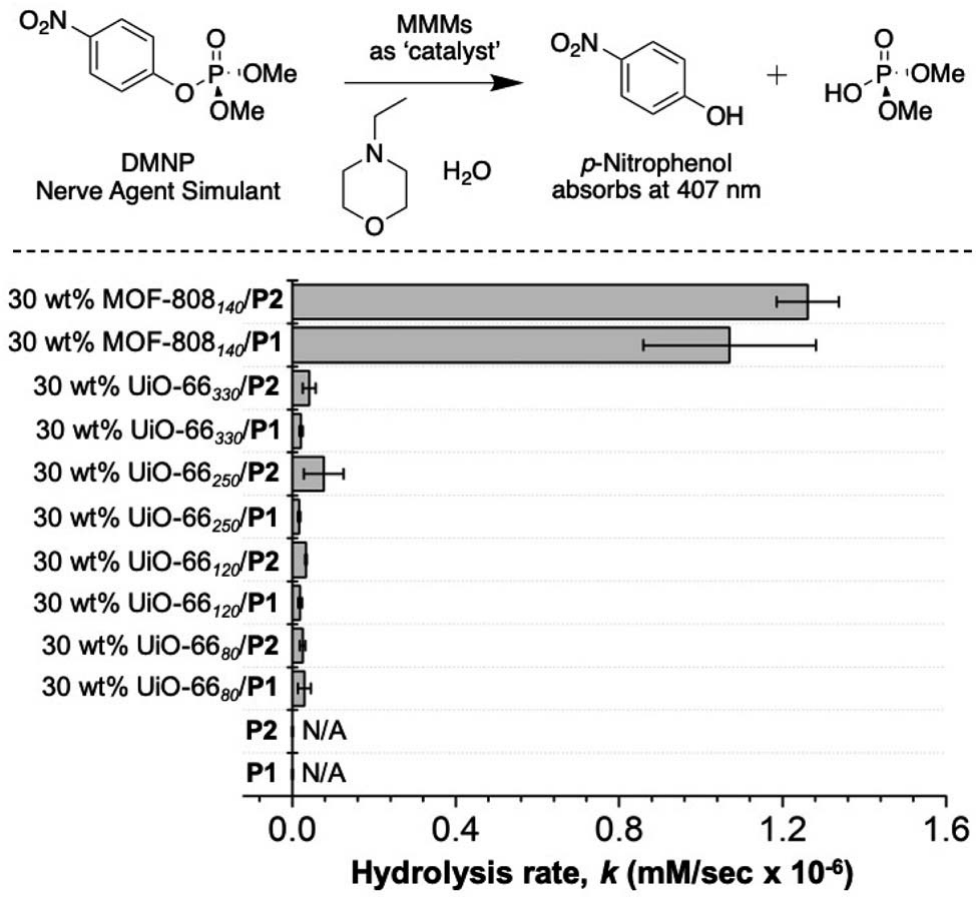
Rate of catalytic degradation of DMNP by MMMs as measured in a UV-Visible adsorption assay (monitored at 407 nm). N/A = no appreciable hydrolysis by P1 and P2.

As shown in Fig. 8, 30 wt% MOF-808_140_/P1 and 30 wt% MOF-808_140_/P2 exhibited ∼10-times better catalytic activity toward the degradation of DMNP than the UiO-66_*X*_-based MMMs. Although dinitrogen gas sorption indicates a lack of porosity at cryogenic temperatures (Table S3†), under the room temperature, solution conditions used for these assays, the catalytic activity suggests that the porosity of the MOF in the MMMs is accessible. Unlike pure P1 and P2, which do not show catalytic activity, the high activity of the corresponding MMMs suggest the MOFs are accessed by DMNP. This is consistent with other literature studies on MOF–polymer composites (including MMMs) that show low porosity *via* cryogenic gas sorption, but good pore access under ambient and solution conditions.^62,63^ Importantly, the activity of the P1-based MMMs remained essentially unaffected after two healing cycles (Fig. S25†).

## Conclusions

In summary, different Zr(iv)-based MOF particles were *in situ* fabricated into MMMs *via* thiol–ene ‘photo-click’ polymerization that exhibited room temperature self-healing behaviour based on reversible boronic-ester hydrolysis chemistry. Tensile analysis showed a considerable improvement in the rigidification of the MMMs while retaining the pristine crystalline structure of MOF. The reversible hydrolysis behaviour of boronic-ester conjugates in water was efficient in making MMMs dynamic and self-healable at room temperature. Interestingly, the mechanical strength of the MMMs increased significantly with the decreasing size of the MOF particles, allowing the possibility to adjust the material properties as a function of particle filler size. The MMMs could degrade the CWA simulant DMNP, and MMM damage, followed by self-healing, had no impact on catalytic activity. Overall, this is the first report of MOF particles in a self-healing MMM, and these materials showed excellent self-healing and CWA degradation performance, which should expand the scope of applications of such MMMs using these dynamic polymer composites. The reported findings offer a strategy to generate healable MMMs under ambient conditions using MOF fillers (and likely other inorganic nanofillers) while maintaining desirable mechanical properties and catalytic reactivity.

## Author contributions

The manuscript was written through the contributions of all authors. P. M. and S. M. C. designed the materials and experimental strategy; P. M. conducted the experiments; P. M. and S. M. C. wrote the manuscript; and P. M. and S. M. C. supervised the editing and writing of this manuscript. All authors have given approval to the final version of the manuscript.

## Conflicts of interest

There are no conflicts to declare.

## Supplementary Material

SC-013-D2SC04345A-s001

SC-013-D2SC04345A-s002
