# Correction: Self-healing mixed matrix membranes containing metal–organic frameworks†

**DOI:** 10.1039/d3sc90156d

**Published:** 2023-08-25

**Authors:** Prantik Mondal, Seth M. Cohen

**Affiliations:** a Department of Chemistry and Biochemistry, University of California La Jolla San Diego California 92093 USA scohen@ucsd.edu

## Abstract

Correction for ‘Self-healing mixed matrix membranes containing metal–organic frameworks’ by Prantik Mondal *et al.*, *Chem. Sci.*, 2022, **13**, 12127–12135, https://doi.org/10.1039/D2SC04345A.

The authors regret that in the original article and supporting information, the chemical structure of the photo-initiator, 2,2-dimethoxy-2-phenylacetophenone (DMP) in Fig. 1 and S5 in the ESI,† was incorrect.

The correct versions of Fig. 1 and S5 are shown here. The original ESI file was replaced with a corrected version on 22/08/2023, and is also available with this Correction article.†

**Fig. 1:**
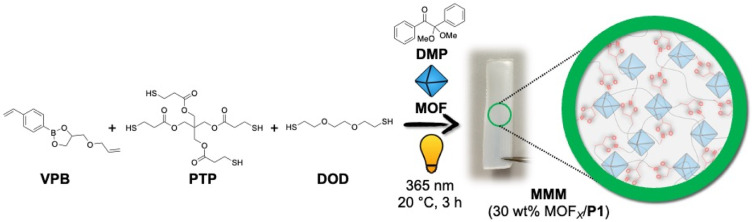
Scheme for synthesizing P1-based MMMs with 30 wt% MOF_*X*_-loading using thiol–ene ‘photo-click’ polymerization.

**Fig. S5** Scheme for synthesizing P2-based (control, not self-healing) MMMs with 30 wt% MOF-loading using thiol–ene ‘photo-click’ polymerization.
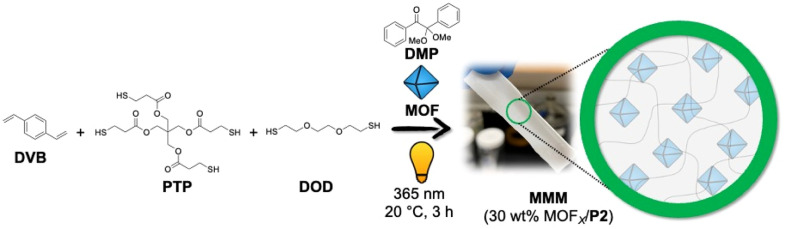


The authors also regret to note that there were errors in Section 2.4 of the supporting information describing the synthesis of UiO-66-NH_2-170_.† The correct version of the Section 2.4 is provided below.

**2.4 Synthesis of UiO-66-NH_2-170_**

In an 8-dram scintillation glass vial with a Teflon-lined cap was placed zirconium(iv) chloride (0.061 g, 2.6 mmol), 2-aminoterephthalic acid (0.047 g, 2.6 mmol), 0.45 mL glacial acetic acid, and 15 mL DMF.. The components were mixed and dissolved by sonicating the reaction mixture for 15 min. The vial was then placed into an isothermal oven and heated at 120 °C for 24 h. After cooling to room temperature, the particles were collected by centrifugation (fixed-angle rotor, 8000 rpm, 5 min), followed by washing with DMF (3 × 20 mL) and methanol (3 × 20 mL). The synthesis and workup procedure were repeated in parallel ten times (*i.e.*, ten individual vials) and the products from all ten reactions were combined and re-suspended in the mixture of 50 mL of methanol, 50 mL of water, and 5 mL conc. HCl. The suspension was then heated to reflux (90 °C) for 18 h. Finally, the particles were again isolated by centrifugation (fixed-angle rotor, 8000 rpm, 5 min), washed with fresh methanol (200 mL), dried under vacuum overnight at 60 °C, and stored in a desiccator until further use.

The Royal Society of Chemistry apologises for these errors and any consequent inconvenience to authors and readers.

## Supplementary Material

SC-014-D3SC90156D-s001

