# Supplementary material for: Correction: Self-healing mixed matrix membranes containing metal–organic frameworks
Source: Chem Sci. 2023 Aug 25;14(36):9978–9. doi: 10.1039/d3sc90156d (PMC10510852; doi:10.1039/d3sc90156d)
Supplement: SC-014-D3SC90156D-s001 [file SC-014-D3SC90156D-s001.pdf]

Electronic Supplementary Material (ESI) for Chemical Science.
This journal is © The Royal Society of Chemistry 2023

Self-healing Mixed Matrix Membranes Containing Metal-Organic Frameworks

Prantik Mondal and Seth M. Cohen*

†*Department of Chemistry and Biochemistry, University of California, San Diego, La Jolla, California 92093, United States*

SUPPORTING INFORMATION

Table of contents

**1. Materials**	S3
**2. Synthetic Procedures**	S3-S8
**3. Characterization details**	S9-S12
**4. Supplementary tables**	S13-S16
**5. Supplementary figures**	S17-S37

1. Materials

The starting materials and solvents related to MOF synthesis were purchased from the chemical suppliers (Sigma-Aldrich, Alfa Aesar, EMD, TCI, and Arkema) and used as received unless otherwise mentioned. Dry dichloromethane (DCM) was obtained from ThermoFisher and used without further purification. 4-Vinylphenylboronic acid (VPB, Combi-Blocks, 98%), 3-allyloxy-1,2- propanediol (TCI, 99%), pentaerythritol tetrakis(3-mercaptopropionate) (PTP, Sigma-Aldrich, 95%), 3,6-dioxa-1,8-octanedithiol (DOD, Sigma-Aldrich, 95%), 2,2-dimethoxy-2-phenylacetophenone (DMP, Sigma-Aldrich, 99%), Dimethyl p-nitrophenylphosphate (DMNP, Sigma-Aldrich, analytical standard), and potassium chloride (BDH, 99%) were used without further purification. Molecular sieves (4 Å, Fisher Scientific) were activated using the Schlenk technique by heating at 120 °C for 2 h under a high vacuum. Normal dimethyl sulfoxide-d_6 (DMSO-d_6, Cambridge Isotope, 99.9% D) was used as received. Dry DMSO-d_6 was prepared by drying the normal DMSO-d_6 overnight over activated 4 Å molecular sieves. Divinylbenzene (DVB, Sigma-Aldrich, 80%) was made inhibitor-free (before polymerization) by passing through a basic alumina column. The UV lamp used for photocuring was a MelodySusie 36 W Nail Lamp equipped with four 9W bulbs and a timer setting of 120 min.

2. Synthetic procedures

2.1 Synthesis of UiO-66$_{330}$

In a 500 mL round-bottomed flask, zirconium (IV) chloride (1.45 g, 6.2 mmol) and terephthalic acid (1.04 g, 6.2 mmol), followed by adding 15 mL glacial acetic acid (262

mmol) and 360 mL of DMF. The mixture was then stirred and sonicated for 30 min to dissolve the reaction components. The mixture was portioned evenly between 24 8-dram vials (with PTFE-lined caps). The vials were then placed in a 120 °C oven for 24 h. After cooling to ambient temperature, the particles were collected by centrifugation (fixed-angle rotor, 8000 rpm, 5 min), followed by washing with 3×200 mL DMF and 3×200 mL methanol. Finally, the particles were dried under vacuum at 60 °C for 24 h to obtain in the form of white-colored microcrystalline powders. The powders were stored in an air-free atmosphere for further use.

2.2 Synthesis of UiO-66$_{160}$

Zirconium (IV) chloride (0.061 g, 0.26 mmol) and terephthalic acid (0.043 g, 0.26 mmol), 15 mL DMF, and 0.45 mL glacial acetic acid were taken in an 8-dram scintillation glass vial with Teflon-lined cap. The components were mixed and dissolved by sonicating the reaction mixture for 15 minutes. The vial was then transferred to an isothermal oven and heated at 120 °C for 24 h. After cooling to room temperature, the particles were collected by centrifugation (fixed-angle rotor, 8000 rpm, 5 min), followed by washing with 3×20 mL DMF and 3×20 mL MeOH. The particles were immersed in methanol for 3 days, during which time the methanol solvent was replaced three times (3×20 mL). After the solvent exchange was completed, the particles were isolated in the form of white-colored powder by centrifuging and decanting the methanol and dried under a vacuum at 50 °C. The particles were stored under air-free conditions.

2.3 Synthesis of UiO-66$_{80}$, UiO-66$_{120}$, UiO-66$_{250}$

UiO-66$_X$ (X = 80 nm, 120 nm, 250 nm) was prepared using a continuous addition method as previously reported (*Angew. Chem. Int. Ed.* **2018**, 57, 7836-7840). The synthesis of UiO-66$_X$ (X = the particle edge length in nm measured by scanning electron microscopy, SEM) at a 5 L scale was carried out at 120 °C under atmospheric pressure in DMF using formic acid as a modulator. Two separate 30 mM stock solutions were prepared in 5L jars. The terephthalic acid (H_2bdc) solution was prepared with 22.5 g of H_2bdc, 4.05 L of DMF, and 450 mL of formic acid, while the $ZrOCl_2·8H_2O$ was prepared with 45 g of $ZrOCl_2·8H_2O$ in 4.5 L of DMF. The reaction procedure is as follows. An initial 100 mL of the $ZrOCl_2·8H_2O$ solution was added to a 5 L round bottom flask at 120 °C, then both the $ZrOCl_2·8H_2O$ and H_2bdc stock solution were separately delivered by a peristaltic pump with a feed rate of 12 mL/min for 5 min. The feed rate was accelerated to 32 mL/min for 55 minutes. After this initial addition, 2.5 L of the reaction solution was removed from the reactor to obtain the first product, UiO-66$_{80}$, and then 1.5 L of metal stock solution and 1.5 L of ligand stock solution were further added into the remaining reaction solution at 30 mL/min for 50 min. Then 3 L of the reaction solution was collected from the reactor to obtain the second product, UiO-66$_{120}$. Finally, 1.55 L of metal stock solution and 1.55 L of ligand stock solution were added into the reactor within 1 h at 25.8 mL/min, and the remaining reaction solution (3.7 L) was collected as the third product UiO-66$_{250}$. All products were first centrifuged (8000 rpm, 30-60 min) and washed with 40 mL DMF twice, and then the solvent exchange was performed by washing 3 times in 40 mL of methanol. The MOF particles were then dried under a vacuum at room temperature and stored under air-free conditions for further use.

2.4 Synthesis of UiO-66-NH_2-*170*

In a 500 mL round-bottomed flask, zirconium (IV) chloride (0.061 g, 2.6 mmol) and 2-aminoterephthalic acid (0.047 g, 2.6 mmol), followed by adding 0.45 mL glacial acetic acid and 15 mL DMF. The mixture was portioned evenly between 10 8-dram vials (with PTFE-lined caps). The components were mixed and dissolved by sonicating the reaction mixture for 15 minutes. The vial was then transferred to an isothermal oven and heated at 120 °C for 24 h. After cooling to room temperature, the particles were collected by centrifugation (fixed-angle rotor, 8000 rpm, 5 min), followed by washing with 3×20 mL DMF and 3×20 mL MeOH. The MOF was resuspended in 200 mL methanol and 20 mL 1M HCl added. The particles were re-suspended in the mixture of methanol and water (1:1 v/v) solvents and 5 mL conc. HCl. The suspension was then heated to reflux (90 °C) for 18 hours. Finally, the particles were isolated by centrifugation, washed with fresh methanol (3×20 mL), and dried under vacuum overnight at 60 °C. The MOF particles were then dried under a vacuum at room temperature and stored under air-free conditions for further use.

2.5 Synthesis of MOF-808*140*

The synthesis of MOF-808 was adapted from a literature procedure (*Chem. Mater.* 2021, 33, 7057-7066). In a 1L thick glass jar, 1,3,5-benzenetricarboxylic acid (H_3btc, 1.49 g, 7 mmol) was taken and dissolved in 128 mL DMF by stirring at 600 rpm. Next, zirconium (IV) oxychloride octahydrate ($ZrOCl_2 \cdot 8H_2O$, 2.16 g, 6.7 mmol) and formic acid (133 mL) were subsequently added under stirring conditions. The reaction mixture was stirred for another 20 min at room temperature. The glass jar was then transferred to an isothermal

oven and heated at 110 °C for 48 h. The white solid was collected by centrifugation (fixed-angle rotor, 8000 rpm, 5 min), washed with 3×240 mL DMF, and re-dispersed in 250 mL DMF. The suspension was then transferred to a 500 mL round-bottomed flask. The suspension was stirred (at 350 rpm) overnight at 100 °C. The solid was then re-isolated by centrifugation (fixed-angle rotor, 8000 rpm, 5 min), followed by washing with 3×240 mL acetone, and re-dispersed in acetone (250 mL). The suspension was again transferred to a 500 mL round-bottomed flask and stirred (at 600 rpm) overnight at 60 °C. Finally, the solid white powder was collected by centrifugation and dried under vacuum at room temperature. The MOF particles were stored under air-free conditions.

2.6 Synthesis of 4-((allyloxy)methyl)-2-(4-vinylphenyl)-1,3,2-dioxaborolane (VPB)

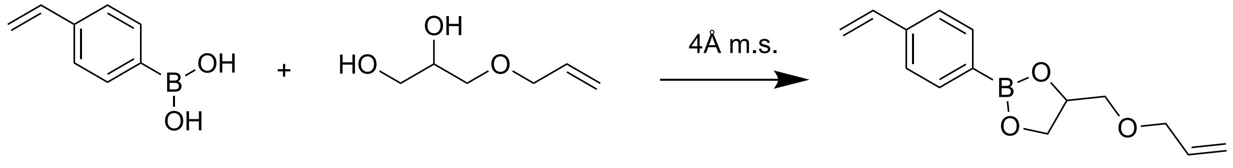

The synthetic protocol of VPB was adapted from a literature procedure (*Macromolecules* 2015, 48, 2098–2106). Initially, 6 g of 4Å molecular sieves (m.s.) were activated under air-free and nitrogenous conditions, applying the Schlenk technique using a J-young flask. Next, the system was cooled to room temperature, followed by the addition of the reaction components, *viz.*, 4-Vinylphenylboronic acid (2.44 g, 16.5 mmol) and 3-allyloxy-1,2-propanediol (1.98 g, 15 mmol), and dry DCM (20 mL). The reaction was then stirred at room temperature for 24 h. After a 12 h reaction, another set of (3.0 g) molecular sieves was added to the reaction system to drive it to completion. Once the reaction was

completed, VPB (in DCM) was filtered and centrifuged. Finally, the supernatant was again filtered and concentrated to give the pale-yellow oil (6 g, 80%). The compound was stored under air-free conditions for further use.

2.7 Fabrication of polymer MMMs

For the preparation of MMMs, all the MOF_X particles were freshly prepared, thoroughly washed and dried, stored under air-free conditions, and used within 1-2 days. Initially, the MOF particles (≈ 0.092 g) were taken in a 1.5-dram glass vial and finely dispersed in 1.2 mL ethyl acetate by sonicating the suspension for 15 min. In a separate 1.5 mL Eppendorf, VPB (0.115 g, 471 μmol), DOD (0.0515 g, 283 μmol), PTP (0.046 g, 94.2 μmol), and DMP (0.0023 g, 9.4 μmol) were taken, followed by vortexing and sonicating the system for 5 min to obtain a clear monomeric mixture. Next, the monomeric mixture was added to the finely dispersed MOF suspension, followed by gentle vortexing it for 60 s. The suspension was then cast on a Teflon mold and immediately shifted to a UV chamber, where the thiol-ene 'photo-click' polymerization was conducted by irradiating the mixture at 365 nm for 3 h. Once the polymerization was completed, the Teflon mold (containing the polymer membrane) was taken out of the UV chamber, cooled to room temperature, and the polymer film was gently peeled off using a spatula. Finally, the polymer material was dried under a high vacuum oven at room temperature for 24 h. Photographs of the mold, UV chamber, and polymer membranes are shown in Figure S6. The divinyl-based (control) polymeric membranes with 30 wt% MOF loading were fabricated using a similar protocol (see Table S1). The polymer films were stored under air-free conditions.

3. Characterization

Nuclear Magnetic Resonance. Proton nuclear magnetic resonance spectra (^1H NMR) were recorded on a Varian FT-NMR spectrometer (400 MHz). Chemical shifts are quoted in parts per million (ppm) referenced to the appropriate solvent peak.

ATR-FTIR analysis. Infrared spectra were collected on dried samples using a Bruker Alpha-P ATR FTIR and analyzed using Opus 6.5 software.

Powder X-Ray Diffraction (PXRD). PXRD data were collected at room temperature on a Bruker D8 Advance diffractometer with a LynxEye XET detector running at 40 kV, 40 mA for Cu Kα (λ = 1.5418 Å), with a scan speed of 1 sec/step, a step size of 0.1° in 2θ, and a 2θ range of 4-40° at room temperature. Sample holders used were zero-background Si plates (p-type, B-doped) from MTI Corp. Well-type sample holders (depth = 0.5 mm) were used for MOF powder samples and polymer films (approximate dimension ≈ 6 mm x 6 mm x 0.1 mm). ~50 mg of dry MOF powder and ~100 mg of polymer samples (~100 mg) mounted on a silicon sample holder were used for analysis by PXRD.

HR-ESI-MS Analysis. High-Resolution Electrospray ionization mass spectrometry (ESI-MS) was performed using a ThermoFinnigan LCQ-DECA mass spectrometer, and the data were analyzed using the Xcalibur software suite. Approximately 5 mg of the sample was digested with sonication in 0.57 mL of DMSO-d_6 and 30 µL of HF (48% aqueous solution).

Thermogravimetric Analysis (TGA). 5-10 mg of MOF powders or MMMs were placed in a 100 µL aluminum crucible. Samples were analyzed on a Mettler Toledo Star TGA/DSC using a temperature range of 30-600 °C, scanning at 10 °C/min under an air atmosphere (75 cm^3/min flow rate) for sample degradation.

Tensile Testing. Tensile strength data were acquired on an Instron® Universal Testing Machine (34SC-05) equipped with a 500N load cell in extension mode. Sample thickness was measured using a Mutityo Digital Micrometer (0-25 mm range, 0.001 mm resolution, IP 54 standard) and averaged from 3 independent measurements from each sample. Tensile measurements were acquired at an extension rate of 10 mm/min with a sampling rate of 500 ms to generate stress-strain curves. Material test specimens were cut with a scissor into rectangular test coupons 6 ± 0.5 mm in width, 10 ± 0.35 mm in length, and 0.12 ± 0.02 mm in thickness. Coupons were clamped vertically with a gauge length of 6 ± 2 mm. Tensile data were collected for at least 3 independent samples.

Scanning Electron Microscopy (SEM). MOF particles or MMM films (approximate dimension ≈ 3 mm x 3 mm x 0.1 mm) were transferred to conductive carbon tape on a sample holder disk and coated using an Ir-sputter coating for 8 seconds. An FEI Apreo SEM instrument was used for acquiring images using a 5 kV energy source under a vacuum at a working distance of 10 mm.

MOF digestion. ~10-20 mg of MOF particles were digested for ^{1}H NMR and mass analyses by solubilizing in 570 µL DMSO-d_6 with 30 µL HF (48% in water).

Humidity Chamber Preparation. Considering the moisture sensitivity of boronic-ester conjugates, the artificial environment with constant 85 % humidity was prepared to minimize the impact of ambient humidity variation during the aging or healing of the membranes (Greenspan et al. *J. Res. Natl. Bur. Stand., Sect. A* **1977,** *81*, 89). The 85% humidity atmosphere was made by inserting a 100 mL thick glass jar containing saturated potassium chloride solution in a sealed 1 L thick glass jar (Figure S26).

Catalysis experiment. The hydrolysis of DMNP using MMMs was conducted in triplicate

using a Synergy HT High-throughput absorbance spectroscopy instrument. The data were generated using Gen 5 v2.09 software, and the values were evaluated and plotted using Excel and Origin software. For the analysis, the polymer membranes were cut into a rectangular-shaped specimen (~12 mm × 8 mm) and placed (along with a 3D-printed plastic holder) in individual wells of an Olympus Plastics clear, flat-bottom 24-well assay plate. The 3D-printed holder is used to keep the membranes from floating to the surface of the well or blocking the beam of the absorbance spectrometer that might generate incorrect and uninterpreted results. The 3D-printed plastic holders are shaped as a hollow triangular prism with one of the three side faces open to the center of the spacer to avoid beam blockage (Figure S27). The 3D-printed holders with a spacer were made in Blender (open-source 3D rendering software, provided upon request as .gcode, .SLDPRT, and .STL files) and printed with a Prusa i3 MK3 3D printer, available at the Digital Media Library of the University of California, San Diego, using polylactic acid (PLA) as the polymer. The polymer MMMs were placed in front of the open spacer of the holder and isolated against the wall of the well. Figure S27 shows how the holder was placed in the 24-well plate with an MMM. A buffer solution (2 mL of 20 mM N-ethylmorpholine in Milli-Q water, pH = 8.0) was added to each well. A solution of dimethyl-4-nitrophenylphosphate (DMNP, 20 µL of 25 mM MeOH) was added immediately before the analysis, and the appearance of *p*-nitrophenoxide was monitored at λ_{max} = 407 nm every 20 s for 60 min. Slopes were calculated from the linear region of each plot (typically at 600−1800 s) and normalized by the mass of the membrane (≈ 25 ± 3 mg).

Self-healing analysis. For the analysis of the healing behavior of the MMMs, material

test specimens were initially cut with a scissor into rectangular test coupons 6 ± 0.5 mm in width, 10 ± 0.35 mm in length, and 0.12 ± 0.02 mm in thickness. Next, the specimens were cut in the middle (of size ≈ 1.8 ± 0.2 mm, using a scissor), followed by dabbing the freshly cut interfaces in a few drops of water for ≈ 15 s. Next, the wet interfaces were reconnected (applying a gentle finger force for ≈ 1 min), placed on a Teflon mold, and stored under ambient conditions for 3 days. Next, the cut-healed samples were dried for 24 h under a vacuum at room temperature. After taking out the cut-healed specimens from the vacuum chamber, their tensile test was immediately performed (within ca. 1-2 min) to assess the healing efficiency quantitatively. An Instron® Universal Testing Machine (34SC-05) was used to evaluate the healing efficiency of the cut-healed specimens.

4. Supplementary Tables

Table S1. Summary of the chemical amounts during the preparation of MMMs.

Boronic-ester-based MMM (**P1**-based MMM)					
Samples	Eq	Amount	Moles	Solvent	Time
VPB	5	0.115 g	0.471 mmol	Ethyl acetate (1.2 mL)	3 h (UV irradiation)
PTP	1	0.046 g	0.094 mmol		
DOD	3	0.051 g	0.283 mmol		
DMP	0.1	0.0025 g	9.4 μmol		
MOF_X	-	0.092 g	-		
Divinyl-based MMM (**P2**-based MMM)					
DVB	5	0.083 g	0.64 mmol	Ethyl acetate (1.2 mL)	3 h (UV irradiation)
PTP	1	0.062 g	0.13 mmol		
DOD	3	0.070 g	0.38 mmol		
DMP	0.1	0.0029 g	0.013 mmol		
MOF_X	-	0.093 g	-		

Table S2. MOF-loading (wt%) in MMMs, as evaluated from TGA.

Polymer	Percent of MOF_X loaded (wt%)
**P1**	0
30 wt% $MOF\text{-}808_{140}$/**P1**	31.8 ± 2.8
30 wt% $UiO\text{-}66_{80}$/**P1**	30.7 ± 0.8
30 wt% $UiO\text{-}66_{120}$/**P1**	29.7 ± 1.3
30 wt% $UiO\text{-}66_{250}$/**P1**	31.6 ± 2.7
30 wt% $UiO\text{-}66_{160}$/**P1**	30.7 ± 1.03
30 wt% $UiO\text{-}66_{330}$/**P1**	32.7 ± 1.9
**P2**	0
30 wt% $MOF\text{-}808_{140}$/**P2**	33.4 ± 2.14
30 wt% $UiO\text{-}66_{80}$/**P2**	32.1 ± 1.05
30 wt% $UiO\text{-}66_{120}$/**P2**	30.5 ± 1.07
30 wt% $UiO\text{-}66_{160}$/**P2**	30.8 ± 0.56
30 wt% $UiO\text{-}66_{250}$/**P2**	29.8 ± 1.37
30 wt% $UiO\text{-}66_{330}$/**P2**	30.2 ± 2.01

Table S3. BET surface area analysis of polymer films and MMMs from three independent samples.

**Material**	**BET surface area (m^2/g)**
UiO-66_{330}	1452 ± 25
MOF-808_{140}	978 ± 10
UiO-66-$NH_{2\text{-}170}$	1264 ± 14
**P1**	1*
30 wt% UiO-66_{330}/**P1**	22 ± 1
30 wt% MOF-808_{140}/**P1**	18 ± 2
**P2**	N/A*
30 wt% UiO-66_{330}/**P2**	20 ± 1
30 wt% MOF-808_{140}/**P1**	14 ± 1

* No appreciable sorption; the standard deviation cannot be calculated.

Table S4. Summary of stress-strain behavior of all polymer materials. Reported values are an average from 3 independent samples.

Polymers	Stress at break (MPa)[a]	Strain at break (%)[a]
**P1**	0.71 ± 0.014	15.8 ± 0.212
30 wt% UiO-66$_{330}$/**P1**	7.74 ± 0.47	29.83 ± 1.31
30 wt% MOF-808$_{140}$/**P1**	16.6 ± 0.56	17.4 ± 0.081
30 wt% UiO-66$_{80}$/**P1**	17.95 ± 0.67	32.51 ± 2.19
30 wt% UiO-66$_{120}$/**P1**	15.05 ± 0.53	17.78 ± 0.32
30 wt% UiO-66$_{160}$/**P1**	13.65 ± 0.38	20.69 ± 1.46
30 wt% UiO-66$_{250}$/**P1**	10.88 ± 1.21	23.02 ± 2.56
**P2**	0.21 ± 0.02	16.68 ± 0.17
30 wt% UiO-66$_{330}$/**P2**	3.61 ± 0.05	21.05 ± 1.57
30 wt% MOF-808$_{140}$/**P2**	5.75 ± 0.36	6.97 ± 0.29
30 wt% UiO-66$_{80}$/**P2**	7.08 ± 0.14	26.73 ± 3.43
30 wt% UiO-66$_{120}$/**P2**	6.20 ± 0.081	16.87 ± 0.87
30 wt% UiO-66$_{160}$/**P2**	4.45 ± 0.29	22.45 ± 1.83
30 wt% UiO-66$_{250}$/**P2**	4.17 ± 0.04	25.02 ± 1.62

[a] Obtained by the uniaxial deformation of the tensile bar until failure (at 10 mm/min strain rate and 20 °C)

5. Supplementary Figures

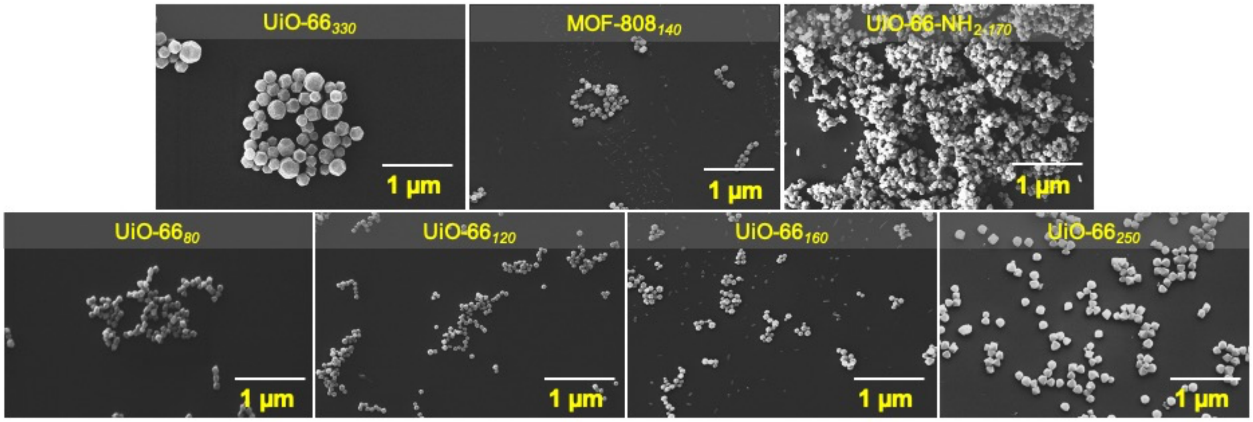

Figure S1. SEM images of MOFs used to prepare MMMs: UiO-66$_{330}$, MOF-808$_{140}$, UiO-66-NH$_{2\text{-}170}$, UiO-66$_{80}$, UiO-66$_{120}$, UiO-66$_{160}$, and UiO-66$_{250}$. (1 μm scale bars).

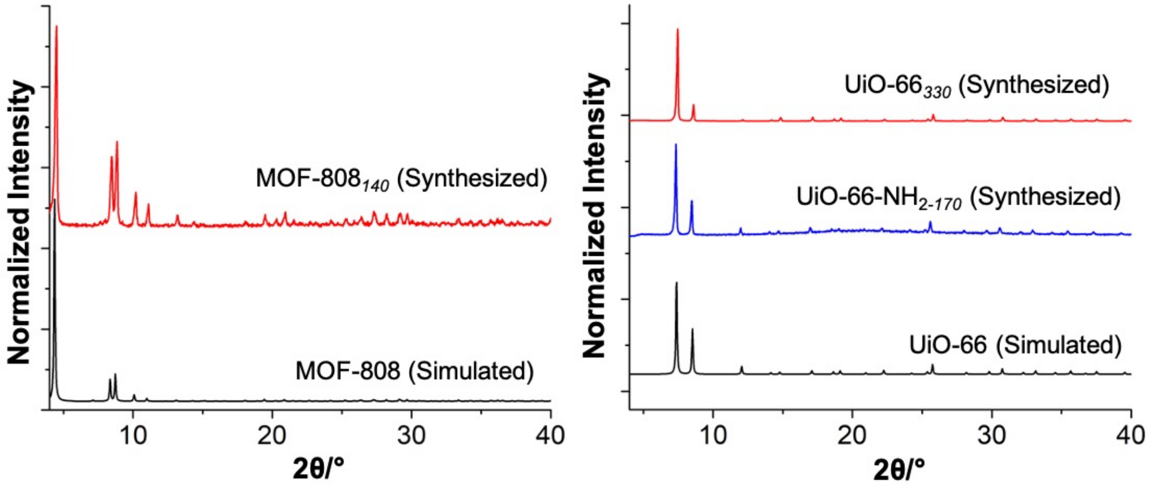

Figure S2. PXRD analysis of MOF-808$_{140}$, UiO-66$_{330}$, and UiO-66-NH$_{2\text{-}170}$.

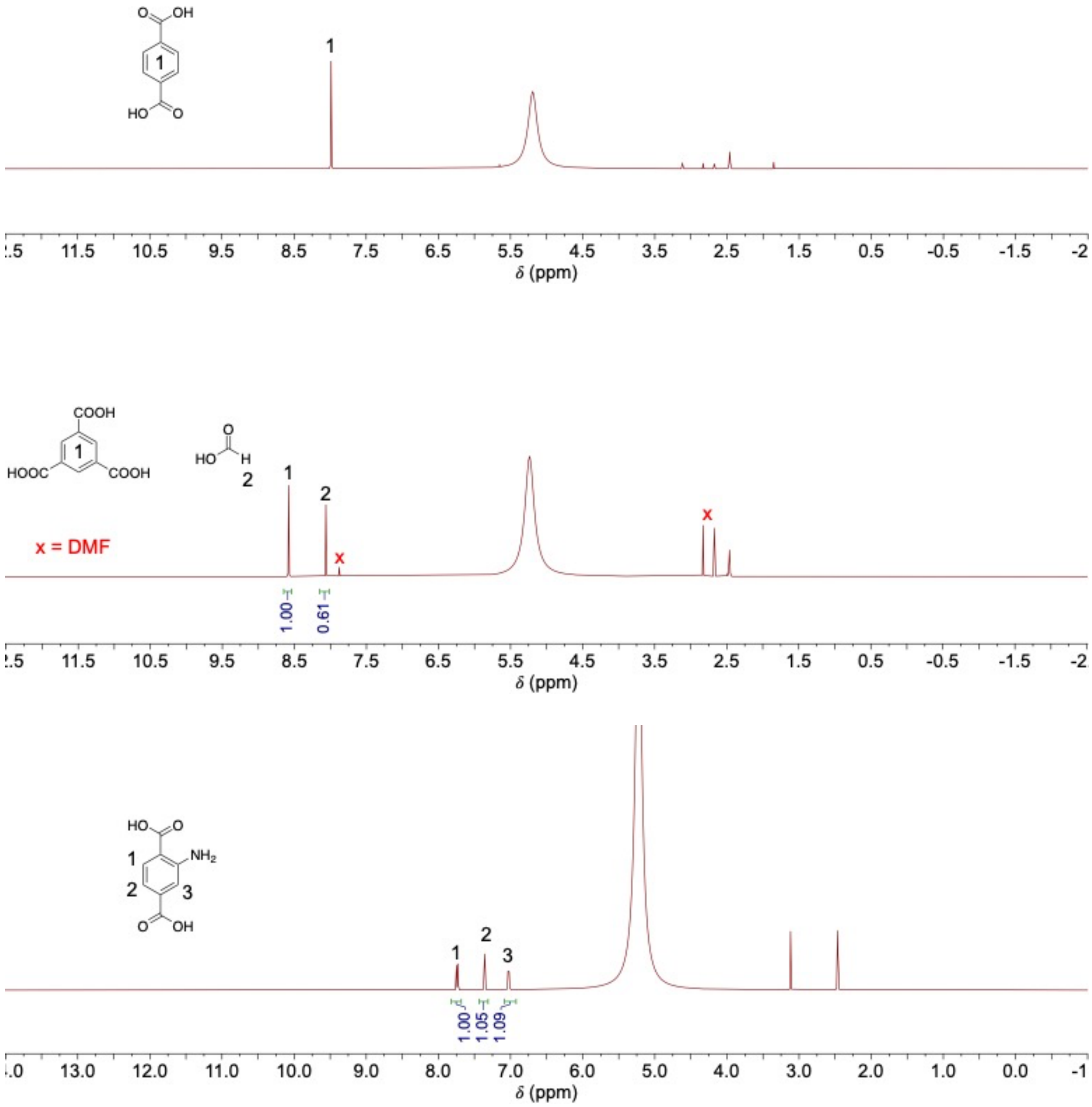

Figure S3. ^{1}H NMR analysis of digested UiO-66$_{330}$ (*top*), MOF-808$_{140}$ (*middle*), and UiO-66-NH$_{2\text{-}170}$ (*bottom*).

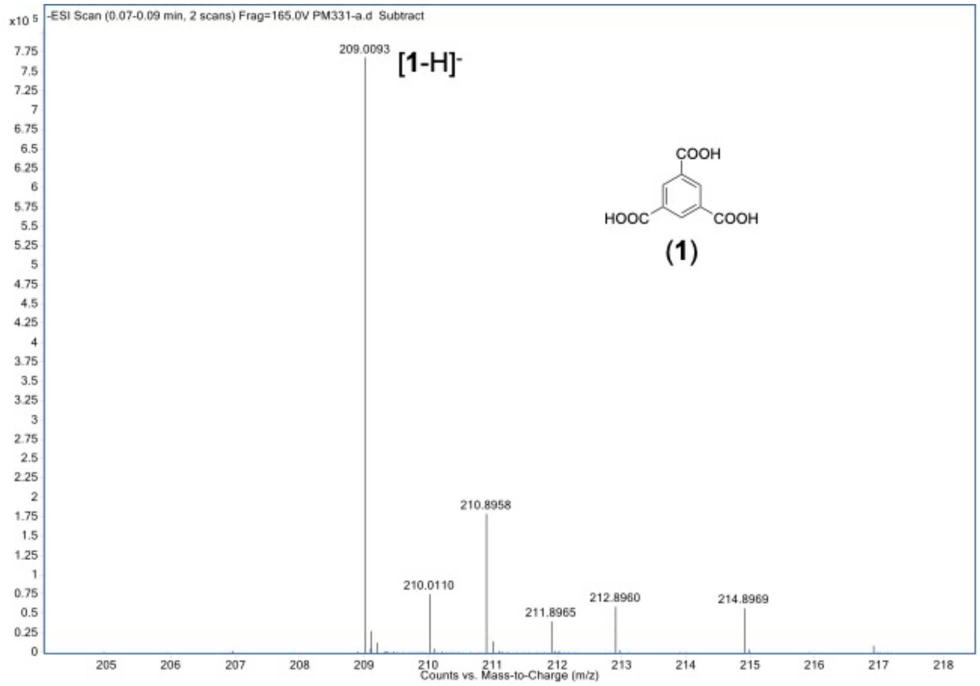

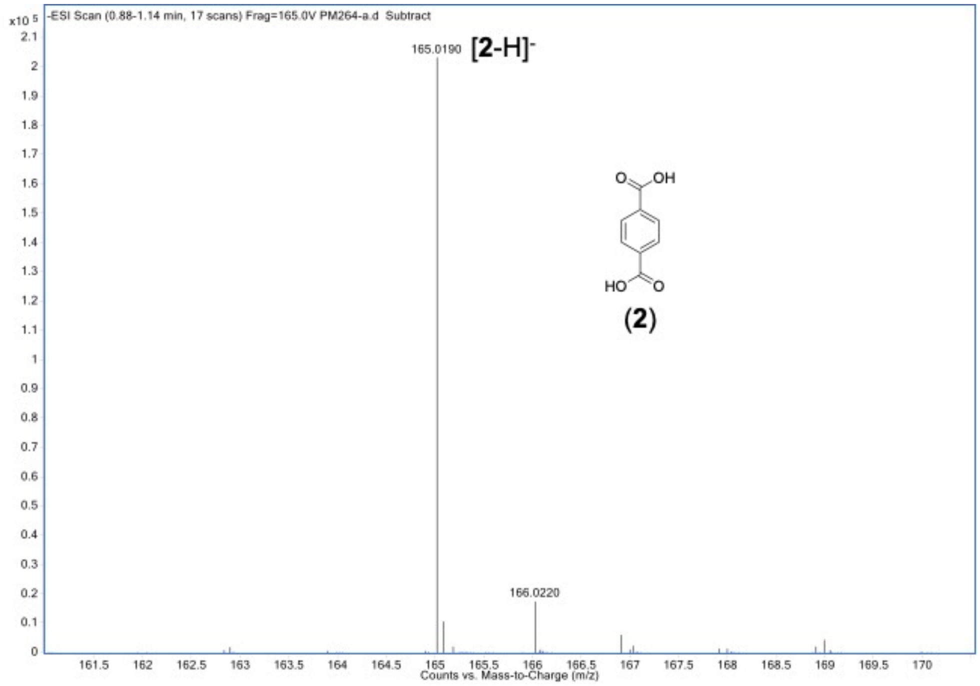

Figure S4. HR-ESI-MS (negative mode) of digested MOF-808$_{140}$ (*top*) and UiO-66$_{330}$ (bottom).

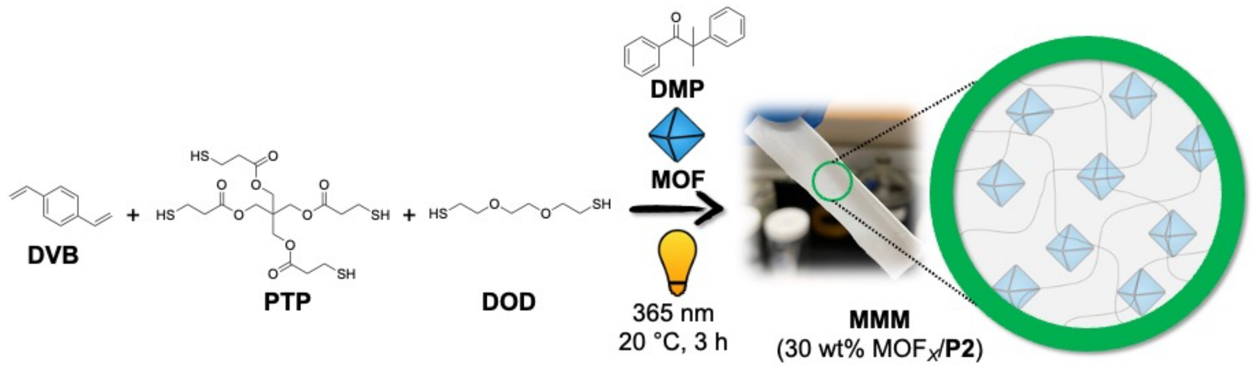

Figure S5. Scheme for synthesizing **P2**-based (control, not self-healing) MMMs with 30 wt% MOF-loading using thiol-ene ‘photo-click’ polymerization.

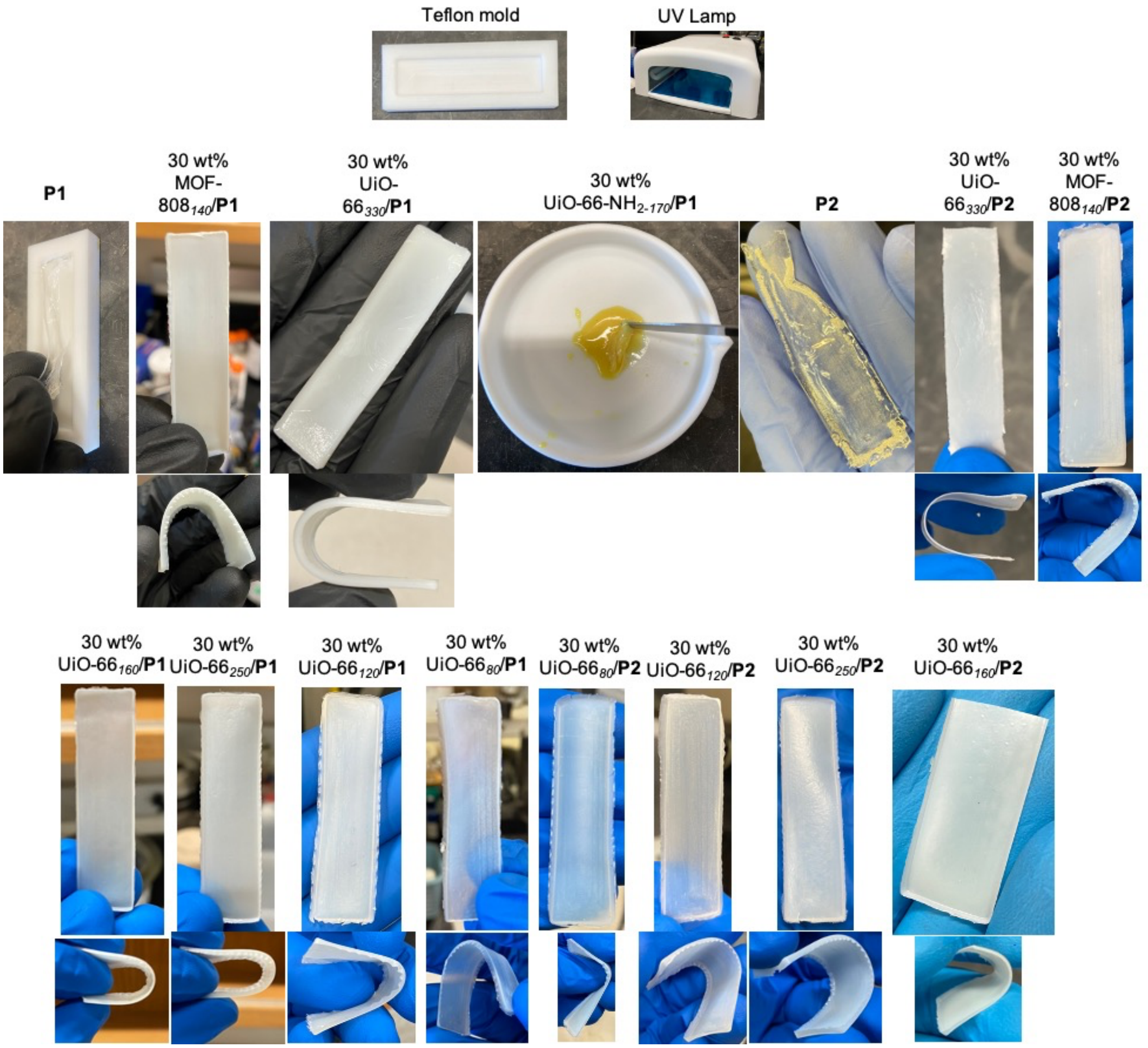

Figure S6. *Top*: Images of the Teflon mold (in which the suspension of MOF and monomer mixture was cast before polymerization) and UV lamp (under which the polymerization was conducted). *Middle and bottom*: Images of the free-standing and flexible MMMs. Pure **P1** and **P2** appeared soft; however, all the MOF-based MMMs were rigid and easily peeled off from the mold. Fabrication using UiO-66-$NH_{2\text{-}170}$ generated a viscous, yellowish mass, indicative of incomplete polymerization.

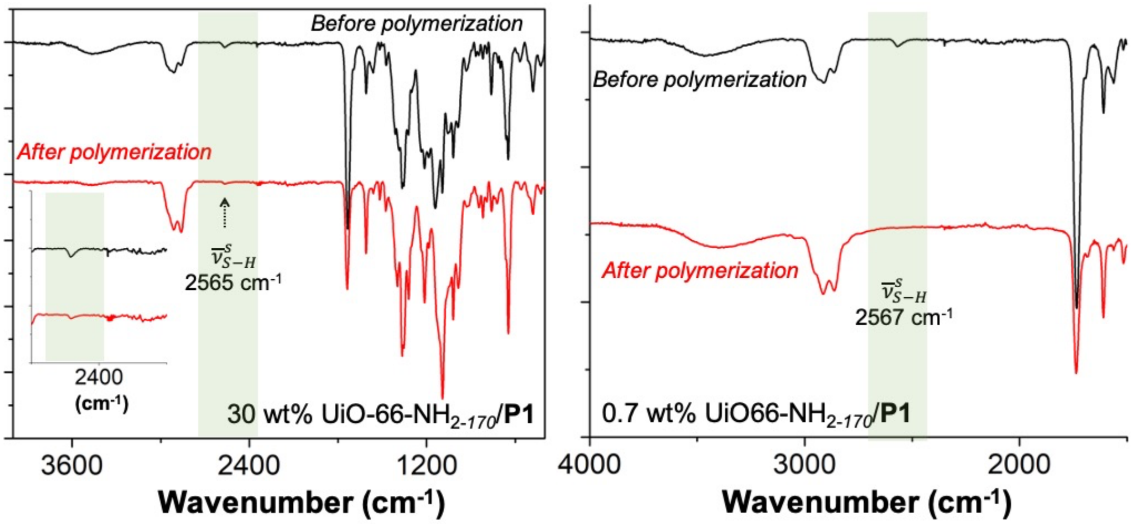

Figure S7. *Left*: FTIR spectrum of 30 wt% UiO-66-$NH_{2\text{-}170}$/**P1**. *Right*: FTIR spectrum of 0.7 wt% UiO-66-$NH_{2\text{-}170}$/**P1**. The green bar is used to highlight the absence of the thiol absorption peak (~2560 cm^{-1}) from the spectra from the corresponding MMMs (after complete polymerization).

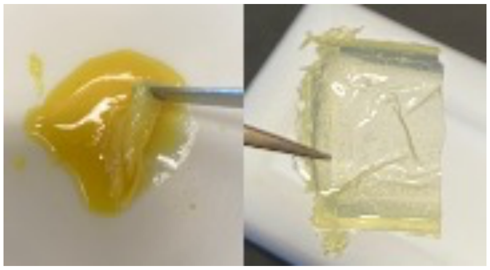

Figure S8. Photographs of MMMs using varied wt% of UiO-66-$NH_{2\text{-}170}$ MOF particles after 3 h polymerization. *Left*: Incomplete polymerization resulting in a viscous mass of 30 wt% UiO-66-$NH_{2\text{-}170}$/**P1**. *Right*: Free-standing membrane of 0.7 wt% UiO-66-$NH_{2\text{-}170}$/**P1** MMM.

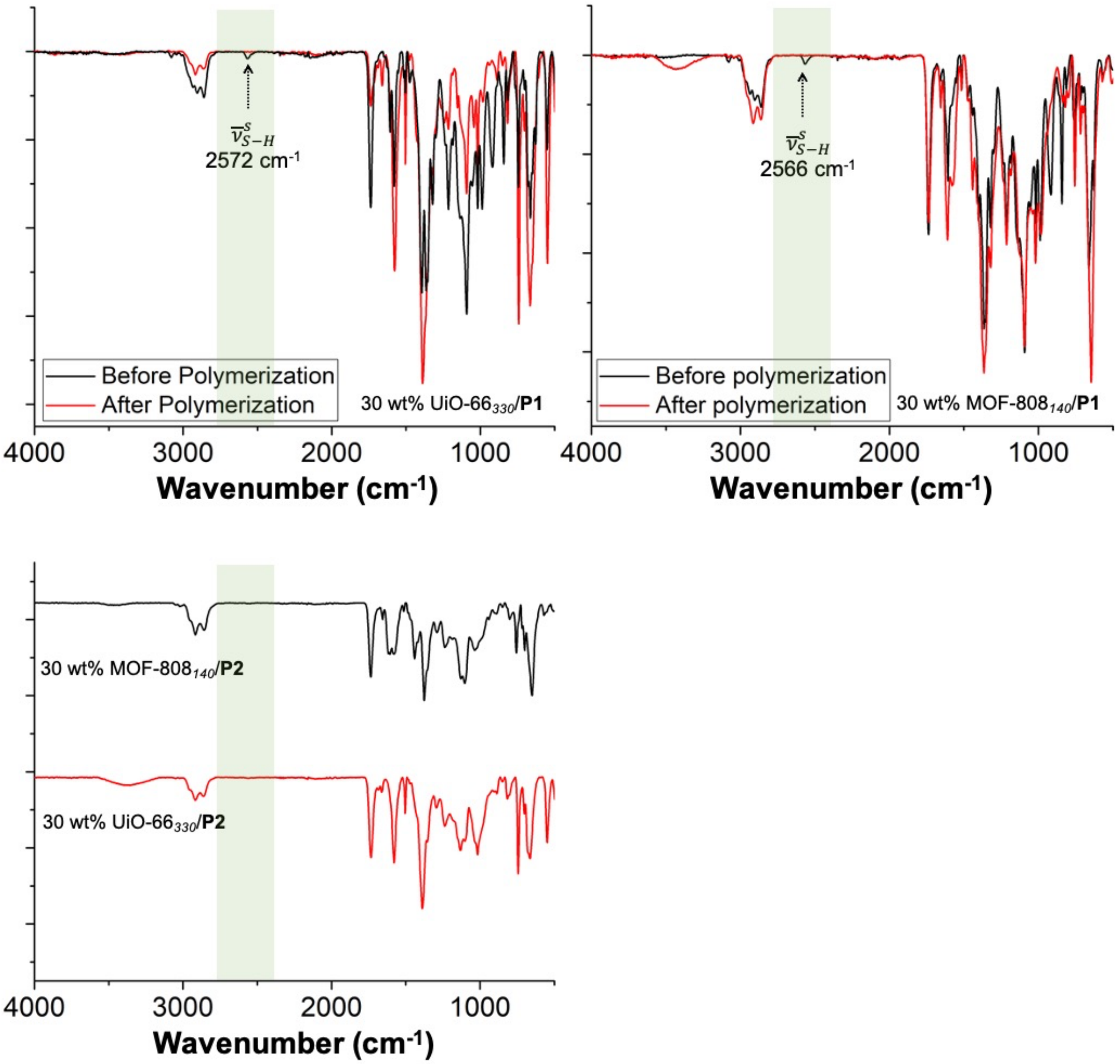

Figure S9. FTIR spectrum of 30 wt% UiO-66$_{330}$/**P1** (*top left*), 30 wt% MOF-808$_{140}$/**P1** (*top right*), and **P2**-based MMMs (*bottom*). The green bar is used to highlight the absence of the thiol absorption peak (~2560 cm^{-1}) from the spectra from the corresponding MMMs (after complete polymerization).

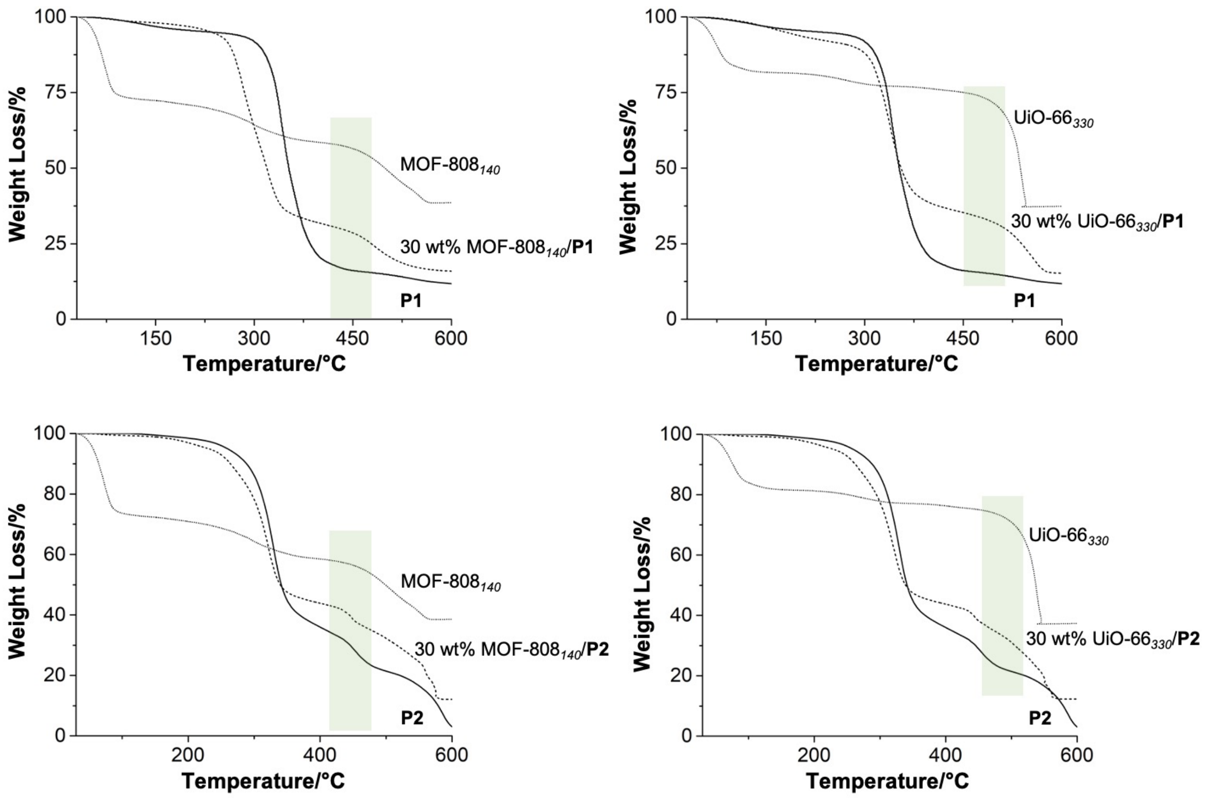

Figure S10. TGA traces of (*top left*) MOF-808$_{140}$, 30 wt% MOF-808$_{140}$/**P1**, and **P1**; (*top right*) UiO-66$_{330}$, 30 wt% UiO-66$_{330}$/**P1**, and **P1**; (*bottom left*) MOF-808$_{140}$, 30 wt% MOF-808$_{140}$/**P2**, and **P2**; (*bottom right*) UiO-66$_{330}$, 30 wt% UiO-66$_{330}$/**P2**, and **P2**. The green bar region is used to calculate the experimental composition of MOF.

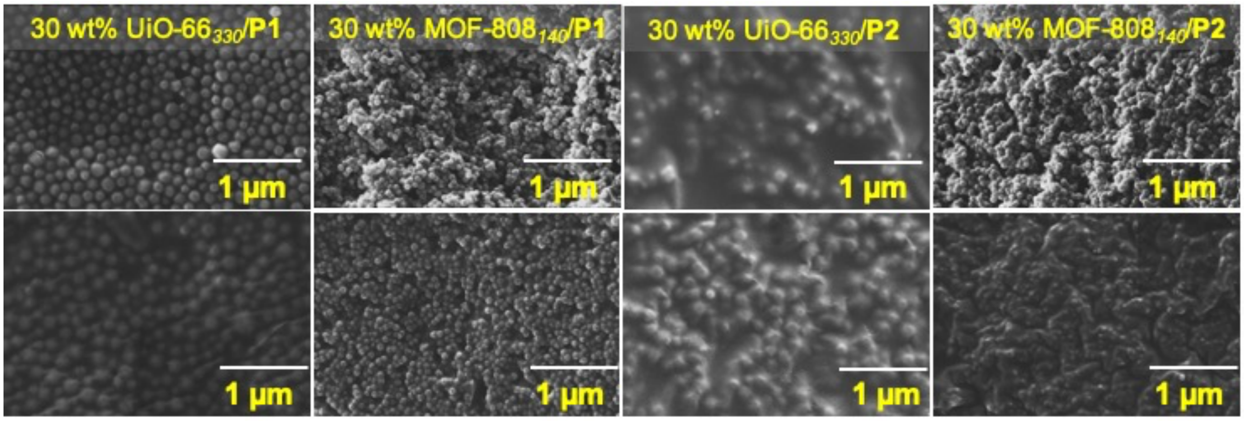

Figure S11. SEM images of the top (*top row*) and bottom (*bottom row*) surfaces of **P1** and **P2**-based MMMs (1 μm scale bar).

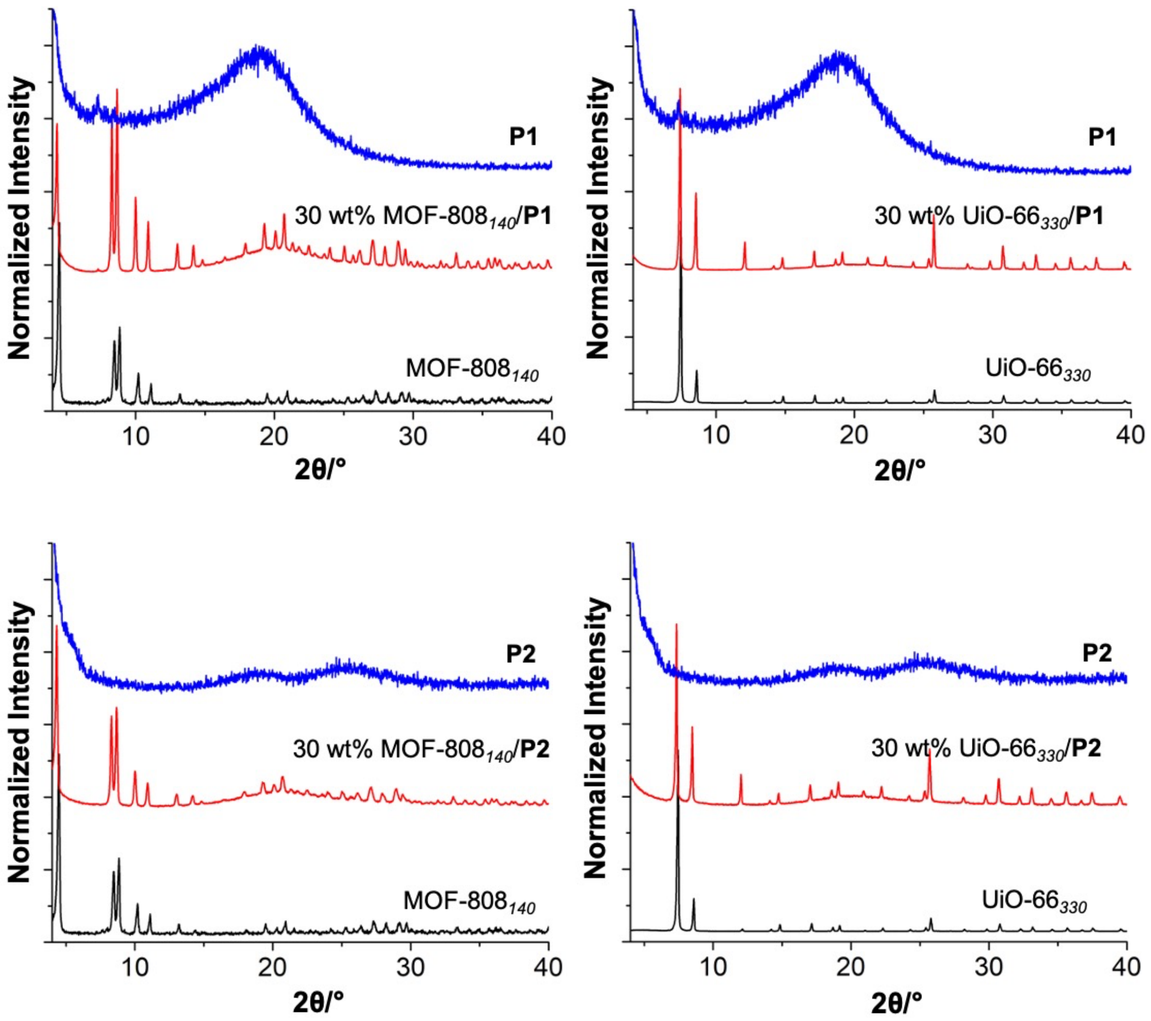

Figure S12. PXRD pattern of (*top left*) **P1**, MOF-808$_{140}$, and 30 wt% MOF-808$_{140}$/**P1**; (*top right*) **P1**, UiO-66$_{330}$, and 30 wt% UiO-66$_{330}$/**P1**; (*bottom left*) **P2**, MOF-808$_{140}$; and 30 wt% MOF-808$_{140}$/**P2**; (*bottom right*) **P2**, UiO-66$_{330}$, and 30 wt% UiO-66$_{330}$/**P2**.

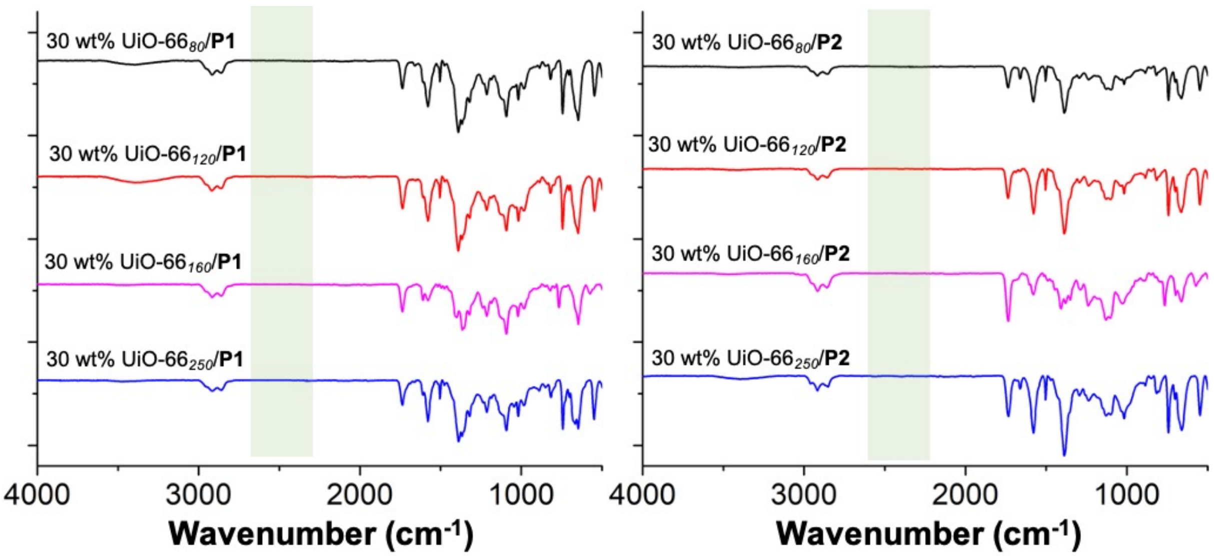

Figure S13. FTIR spectrum of **P1**-based (*left*) and **P2**-based (*right*) MMMs. The green bar is used to highlight the absence of the thiol absorption peak (~2560 cm^{-1}) from the spectra from the corresponding MMMs (after complete polymerization).

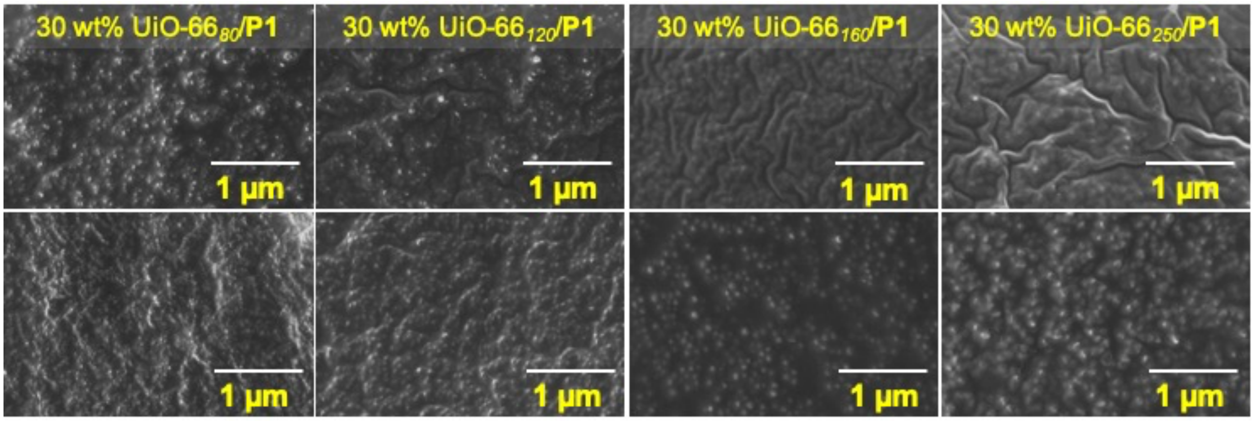

Figure S14. SEM images of (*top row*) top and (*bottom row*) bottom surfaces of **P1**-based MMMs.

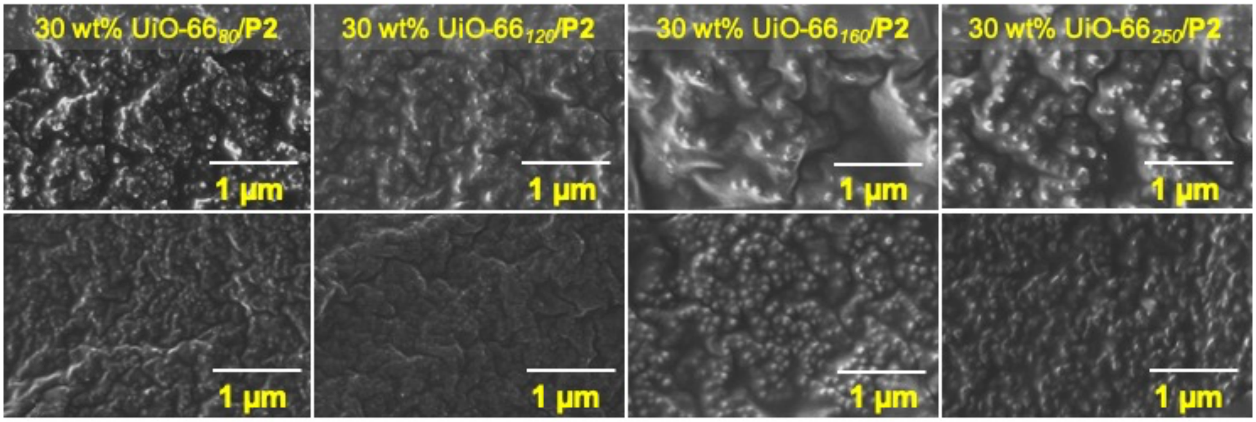

Figure S15. SEM images of (*top row*) top and (*bottom row*) bottom surfaces of **P2**-based MMMs.

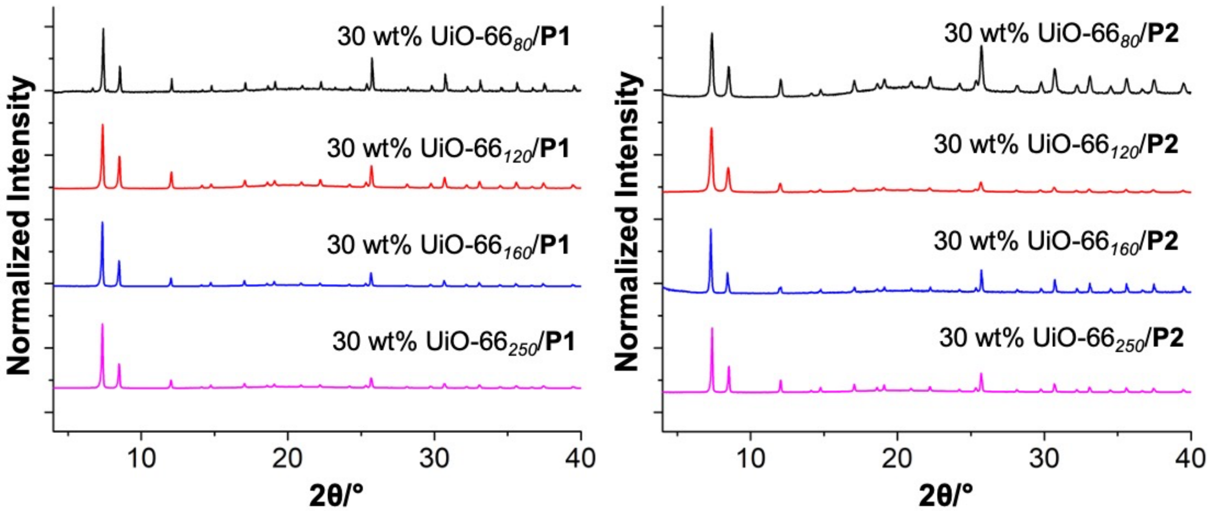

Figure S16. PXRD pattern of **P1**-based (*left*) and **P2**-based (*right*) MMMs.

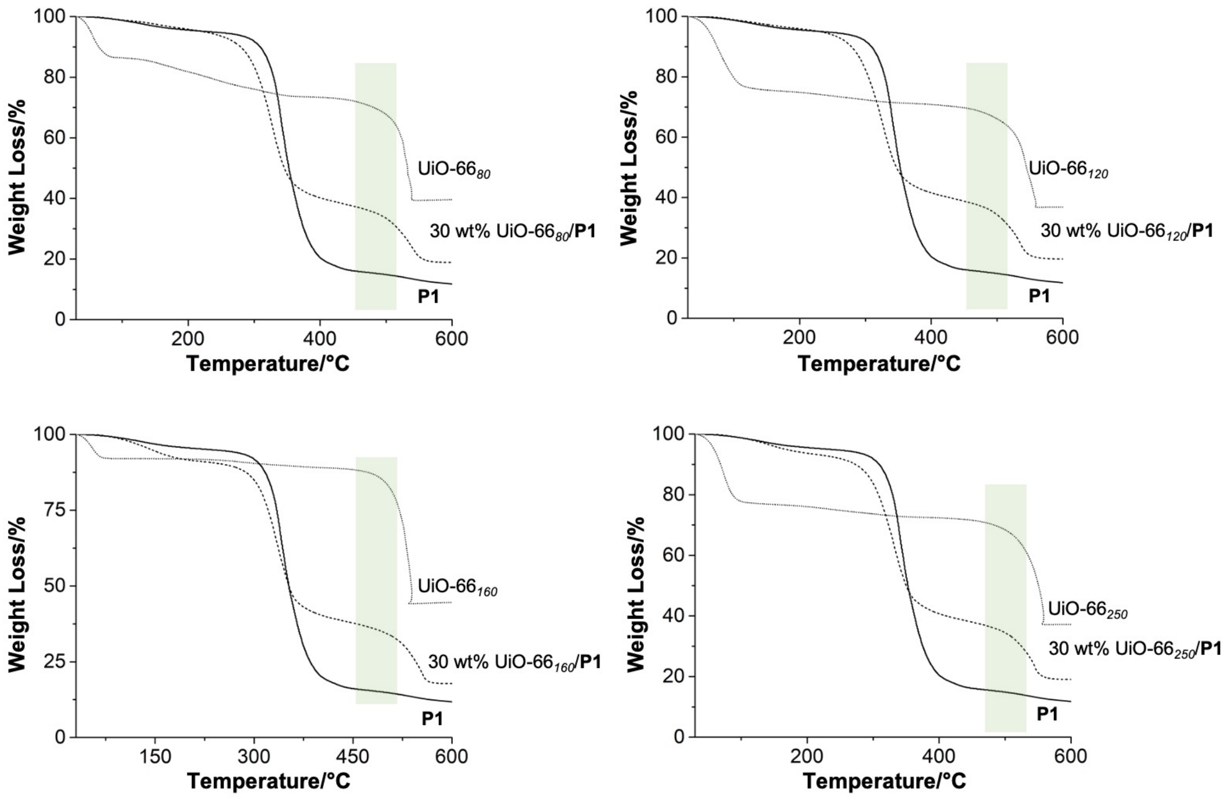

Figure S17. TGA of (*top left*) UiO-66$_{80}$, 30 wt% UiO-66$_{80}$/**P1**, and **P1**; (*top right*) UiO-66$_{120}$, 30 wt% UiO-66$_{120}$/**P1**, and **P1**; (*bottom left*) UiO-66$_{160}$, 30 wt% UiO-66$_{160}$/**P1**, and **P1**; (*bottom right*) UiO-66$_{250}$, 30 wt% UiO-66$_{250}$/**P1**, and **P1**. The green bar region is used to calculate the experimental composition of MOF.

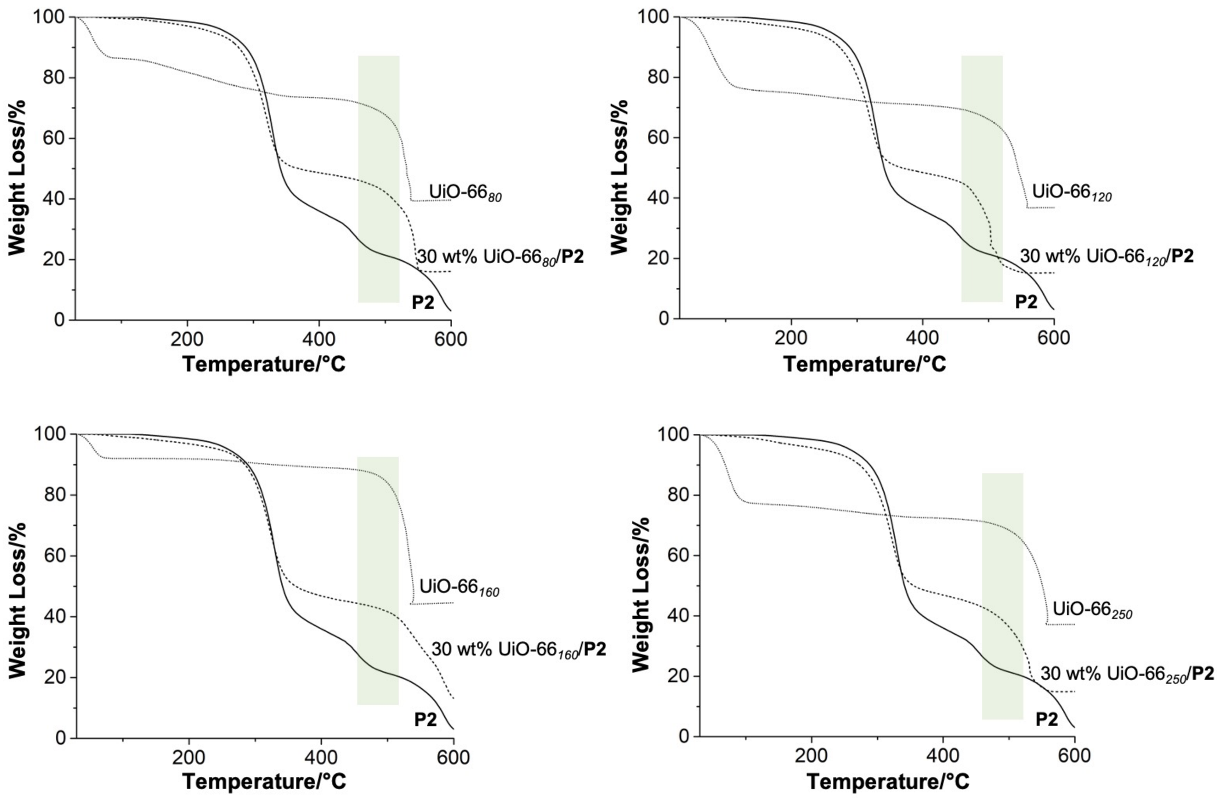

Figure S18. TGA trace of (*top left*) UiO-66$_{80}$, 30 wt% UiO-66$_{80}$/**P2**, and **P2**; (*top right*) UiO-66$_{120}$, 30 wt% UiO-66$_{120}$/**P2**, and **P2**; (*bottom left*) UiO-66$_{160}$, 30 wt% UiO-66$_{160}$/**P2**, and **P2**; (*bottom right*) UiO-66$_{250}$, 30 wt% UiO-66$_{250}$/**P2**, and **P2**. The green bar region is used to calculate the experimental composition of MOF.

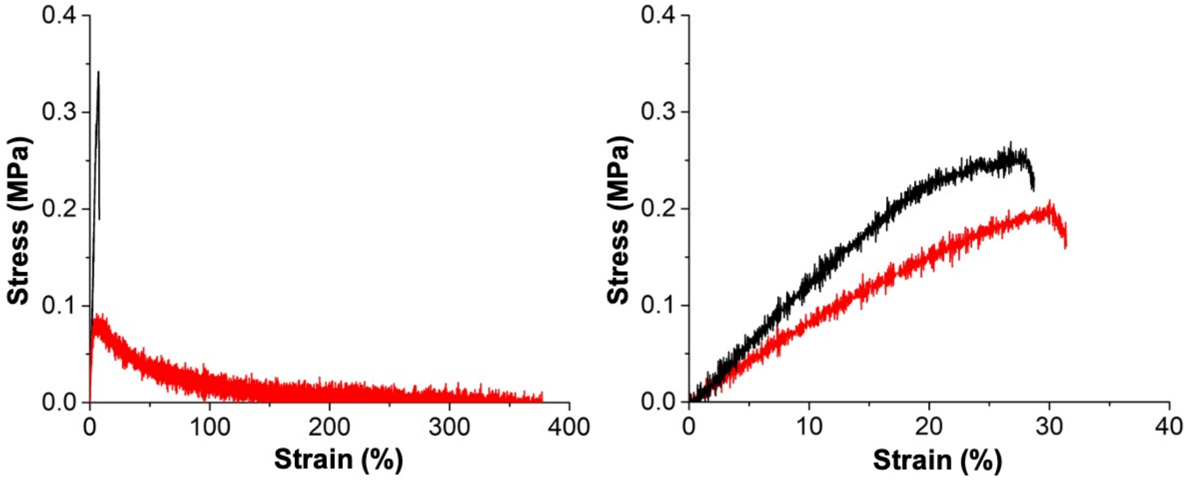

Figure S19. Measurements highlighting the dynamic behavior of **P1** (*left*) and **P2** (*right*) via alteration in tensile properties. Black traces are the original samples. Red traces are after being aged at 85% humidity for 24 h.

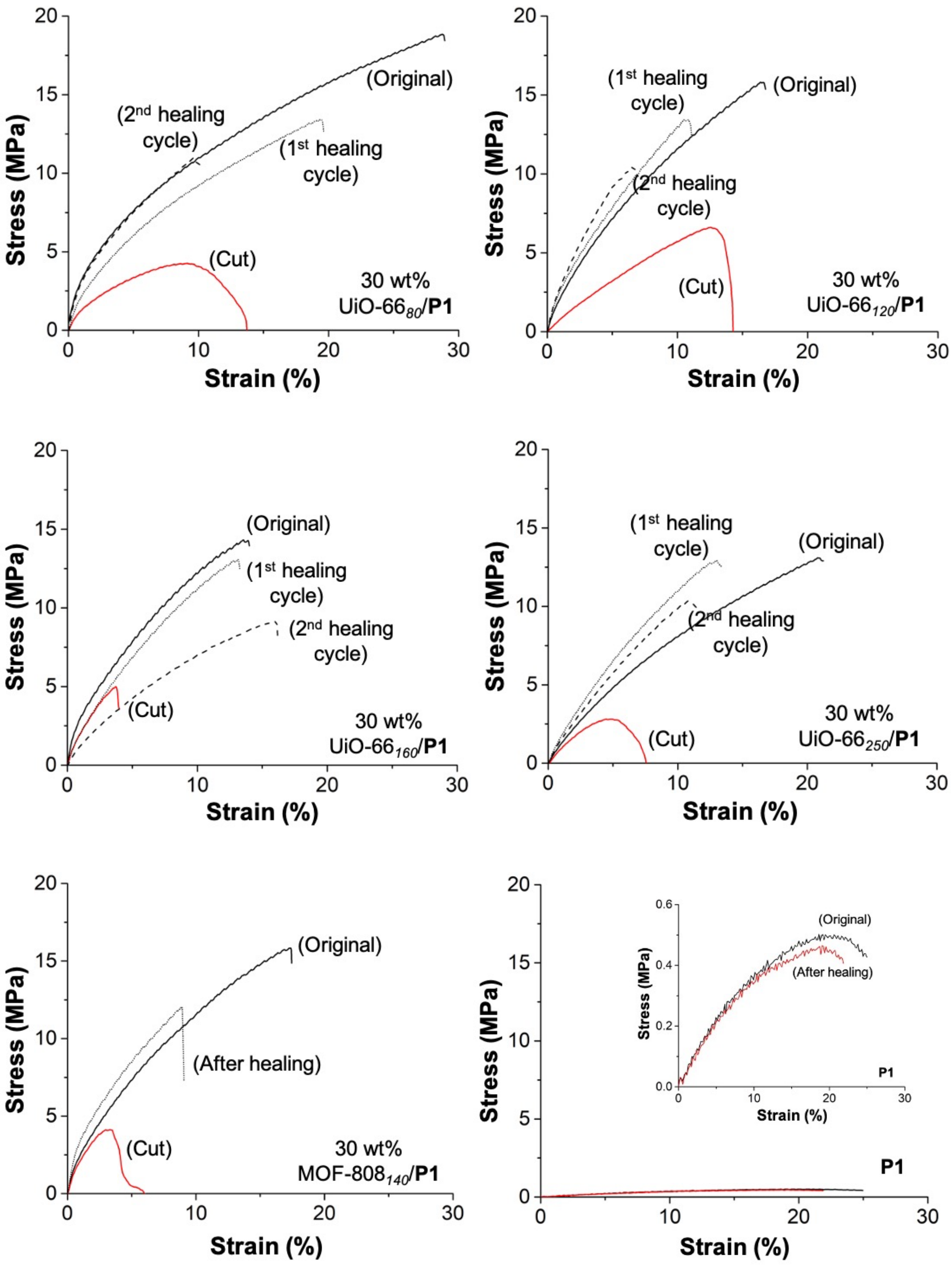

Figure S20. Stress-strain behavior of cut and self-healed: 30 wt% UiO-66$_{80}$/**P1**, 30 wt% UiO-66$_{120}$/**P1**, 30 wt% UiO-66$_{160}$/**P1**, 30 wt% UiO-66$_{250}$/**P1**, 30 wt% MOF-808$_{140}$/**P1**, and **P1** (with inset provided for clarity due to a shallow stress-strain curve).

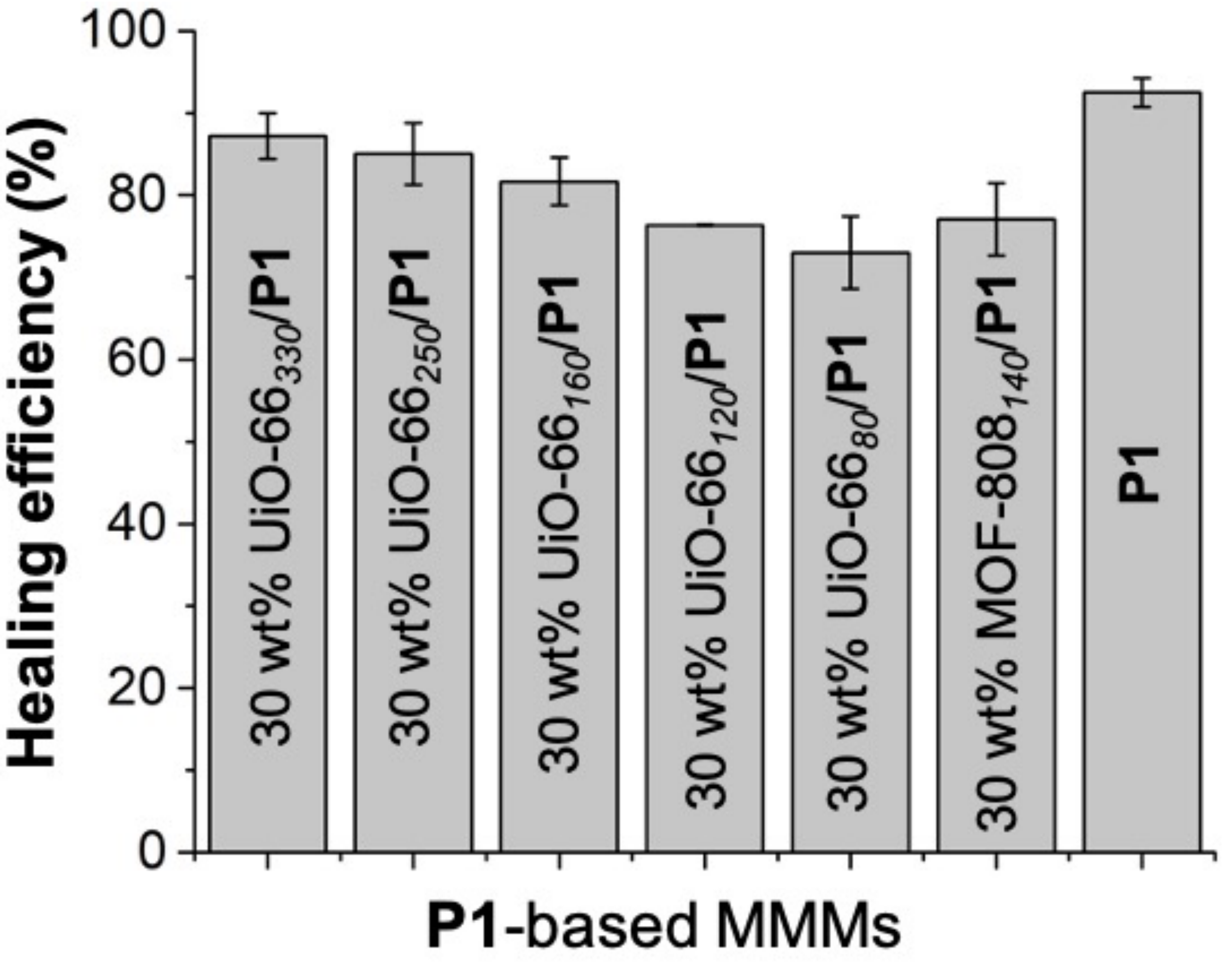

Figure S21. Healing efficiency (%) of **P1** and **P1**-based MMMs. The healing efficiency of MMMs was determined based on the recovery of tensile strength (σ_b) for three independent samples. Healing efficiency is typically defined as the ratio of mechanical strength of healed and virgin materials (White et al. *Nature* **2002**, *409*, 794-797). Here, the healing efficiency was calculated using tensile strength and the following formula (adapted from Klumperman et al. *Macromolecules* **2011,** *44*, 2536-2541):

$$Healing\ efficiency\ (\%) = \frac{\sigma_b\ of\ healed\ MMM}{Original\ \sigma_b\ of\ MMM} \times 100$$

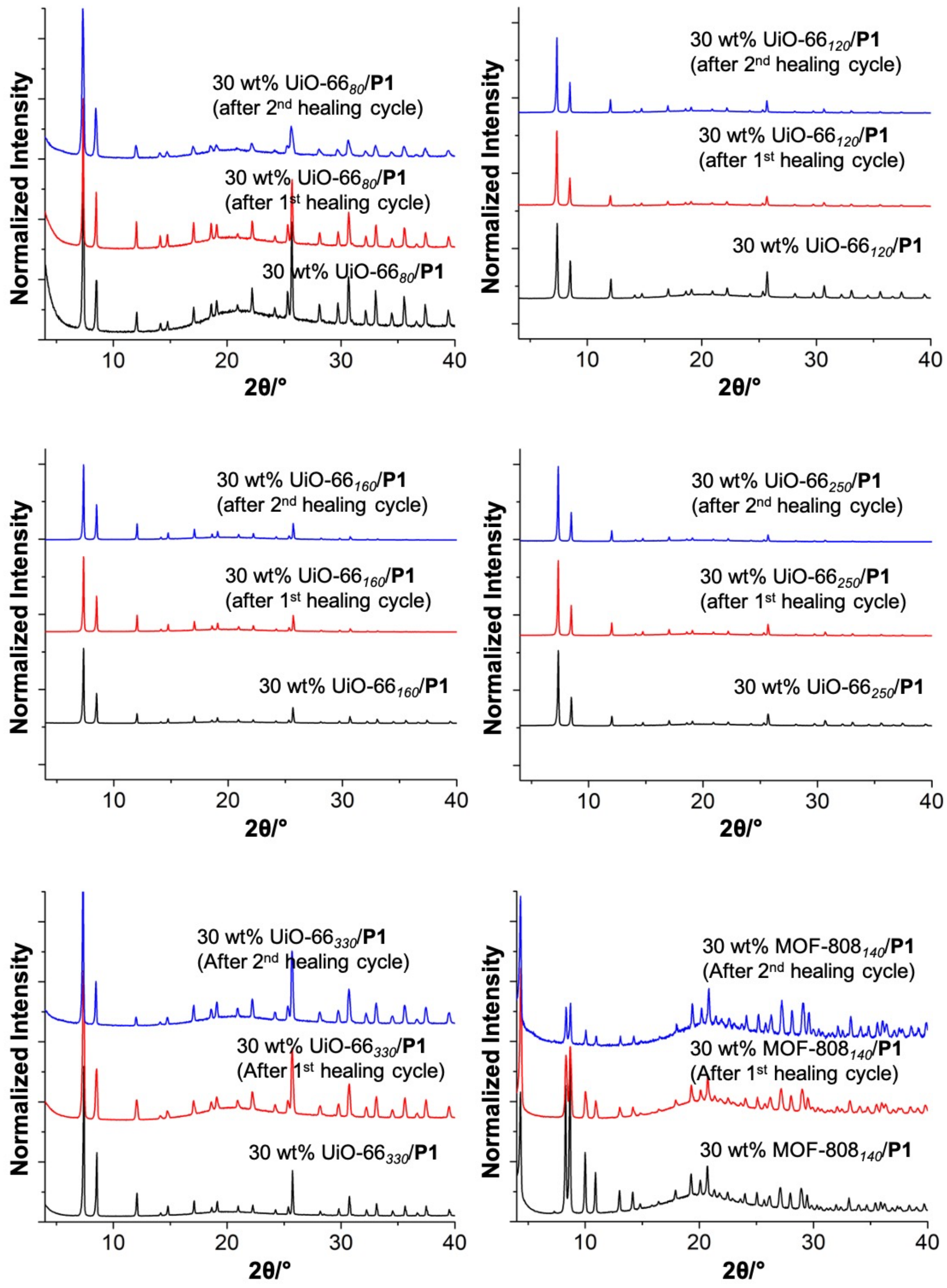

Figure S22. PXRD pattern of original **P1**-based MMMs and after two consecutive healing cycles.

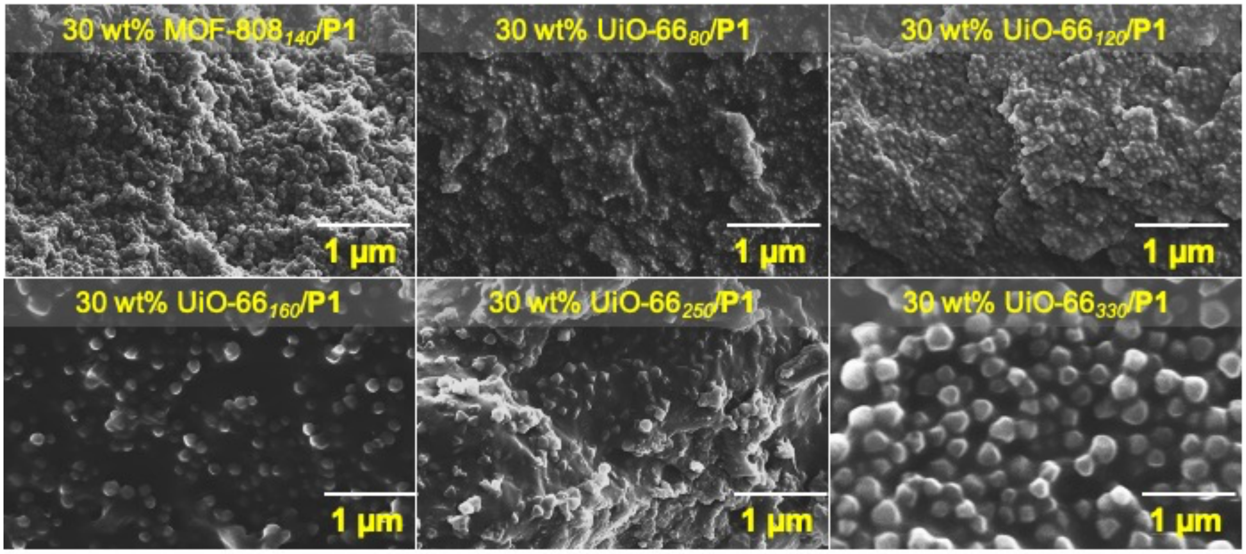

Figure S23. SEM images of the cross-sectional surface of **P1**-based MMMs after two consecutive healing cycles (1 μm scale bar).

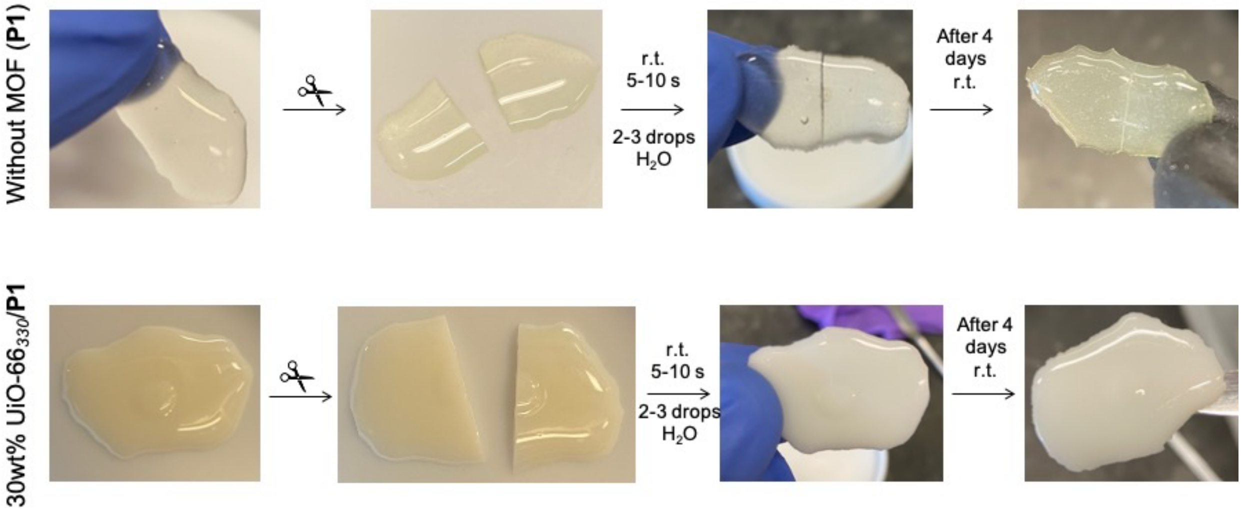

Figure S24. Macroscopic self-healing illustration of 30 wt% UiO-66$_{330}$/**P1** (see Supporting Movie S1 for a more complete record of the entire healing process).

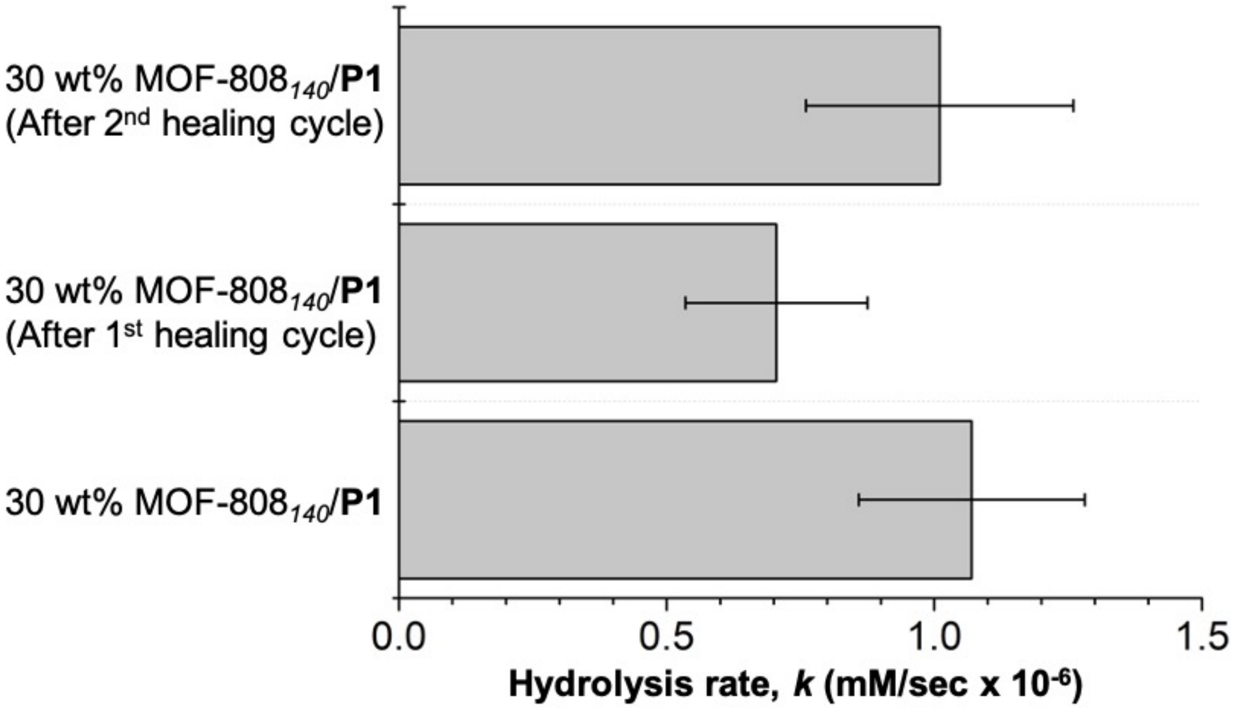

Figure S25. Rate of catalytic degradation of DMNP by pristine 30 wt% MOF-808$_{140}$/**P1** and after healing cycles, as measured in a UV-visible adsorption assay.

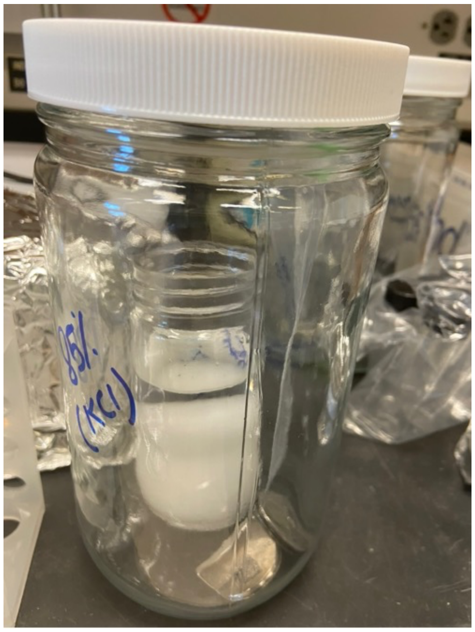

Figure S26. Artificial 85% humidity chamber.

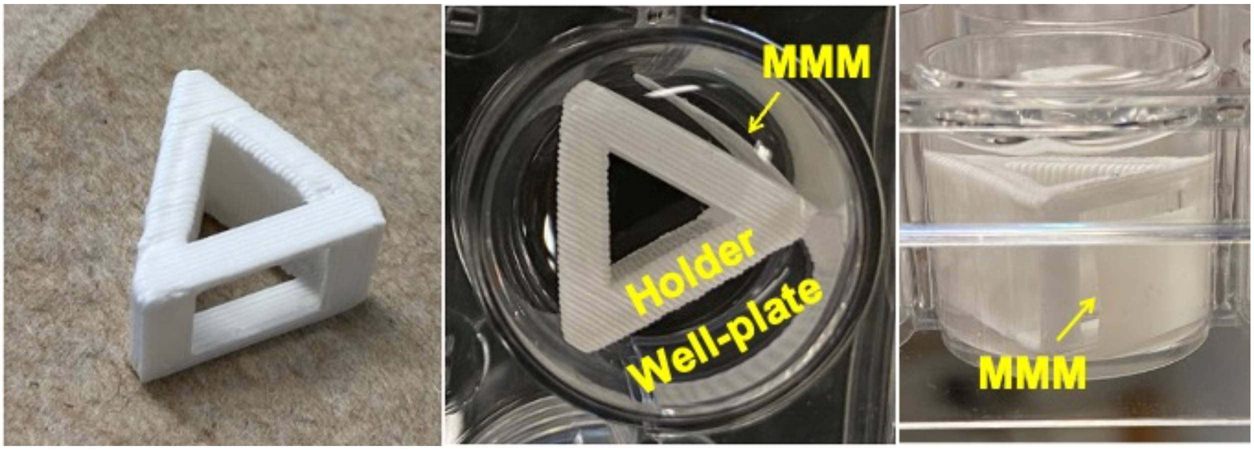

Figure S27. Photographs of (*left*) 3D printed plastic 'holder' and photographs of the top-view (*left*) and cross-sectional view (*right*) of a well containing a 3D-printed holder with an MMM in 2 mL of 20 mM *N*-ethylmorpholine in Milli-Q water (pH = 8.0). The MMM was placed in front of the open spacer of the holder and isolated against the wall of the well to prevent it from interfering with the UV-visible measurement without limiting solution access to its surfaces.
